# Supplementary material for: SIMBA: single-cell embedding along with features
Source: Nat Methods. 2023 May 29;21(6):1003–13. doi: 10.1038/s41592-023-01899-8 (PMC11166568; doi:10.1038/s41592-023-01899-8)
Supplement: Supplementary file 1 — Supplementary Figs. 1–28, Supplementary Notes 1–9 and Supplementary Table 1. [file 41592_2023_1899_MOESM1_ESM.pdf]

nature methods

Article https://doi.org/10.1038/s41592-023-01899-8

SIMBA: single-cell embedding along with features

In the format provided by the authors and unedited

Supplementary Notes

Supplementary Note 1: Comparson of SIMBA with existing embedding methods

Although there are recent proposals for single-cell embedding methods [1-3] , the core algorithm and graph construction procedure underlying SIMBA as well as the resulting embedding and downstream applications offer unique advantages. GLUE[1], which performs integration on unpaired single-cell multi-omic data, embeds cells into a low-dimension manifold using a variational autoencoder (VAE).This procedure is guided by a knowledge-based graph of omic features as nodes. It outputs embeddings of cells and features, however these embeddings are not comparable. GLUE is unapplicable to use-cases outside of unpaired multi-modal analyses, including single-modality analyses, making it a tool with a very specific use-case. Metacell[2] is a scRNA-seq analysis method that uses a k-nearest neighbor (kNN) graph with node featurization to merge highly similar cells into 'metacells'. It is applicable to only scRNA-seq data and the resulting embedding contains only cells. scETM[3] is a generative topic model for integrative analysis of scRNA-seq data. It learns embeddings of cells and genes through VAE. Similar to GLUE, its resulting embeddings are not comparable. Similar to Metacell, it can only be applied to scRNA-seq data. CellSpace [4] is a scATAC-seq embedding method based on TagSpace, a non-graph-based model from StarSpace[5]. In this model sampled DNA sequences are represented as sentences with k-mers treated as words; cells are represented as hashtags. CellSpace is designed solely for scATAC-seq data, and the resulting embeddings contain cells and a single-type feature, i.e., DNA sequences. MIRA [6] is a multimodal analysis method that uses a VAE to learn embeddings of cells by concatenating low-dimensional representations of cells from scRNA-seq and scATAC-seq data. MIRA is designed for only single-cell multimodal data and the resulting embeddings contain only cells. Compared to existing methods and summarized in **Supplementary Table 1**, several key innovations and advantages of SIMBA are most notably highlighted by (1) SIMBA does not require node featurization nor ad-hoc transformations. (2) The co-embedding of cells and features in a common space generated by SIMBA enables an intuitive interface for inferring biologically meaningful information (e.g., marker genes, regulators, cell circuits). (3) While SIMBA is not a dedicated method for specific single-cell analysis tasks, it is able to perform better or comparably well than existing state-of-the-art methods that are dedicated to respective tasks. Finally, (4) SIMBA is implemented as a high-quality, comprehensive, and well-documented Python package built on the Anndata structure and compatible with, Scanpy – the most widely-used Python-based single-cell analysis framework, making it readily accessible to a wide audience.

Supplementary Note 2: Quantitative validation of marker features identified by SIMBA

To quantitatively evaluate embeddings generated by SIMBA, we provide a Gini-index-based quantitative procedure to validate the marker features identified by SIMBA. We reasoned that a good marker feature is specifically expressed in one or a few cell types and therefore has relatively high inequality, i.e., a higher Gini index, across cell types. We have applied this

procedure to 3 datasets: the 10x PBMCs scRNA-seq dataset, the human hematopoiesis scATAC-seq dataset, and the mouse skin SHARE-seq multiome dataset.

These datasets provide well-annotated cell types that can be used to assess quantitatively the quality of the recovered marker and it is worth noticing that SIMBA never used this information to learn the embeddings or detect the markers. In fact, SIMBA does not need cell type or cluster labels to detect marker features.

For each dataset we first pick the top marker features identified by SIMBA (top 500 marker genes for scRNA-seq; top 5,000 marker peaks for scATAC-seq). For each marker feature, we then calculated its average expression or accessibility level for each cell type (top marker features are shown in the dot plots as shown in Supplementary Fig. 5). We then calculate the Gini index scores for these marker features and compared them with the "background" features (for gene expression, "background" genes consist of one group of 500 randomly selected genes and one group of house-keeping genes; for chromatin accessibility, "background" peaks consist of 5,000 randomly selected peaks).

As shown in Supplementary Fig. 5, the Gini index scores of the marker features identified by SIMBA are significantly higher than "background" features (p-value < 10-4, Mann-Whitney-Wilcoxon test) for all four cases.

Supplementary Note 3: Comparison of SIMBA in marker gene detection

We compared the top 600 marker genes identified by SIMBA (based on max value and Gini index) with those identified by the clustering-based statistical-tests method implemented in Scanpy (based on z-score calculated from the two-sided Wilcoxon rank-sum test with a Benjamini-Hochberg p-value correction, one of the statistical tests recommended in Scanpy's tutorial) (**Supplementary Fig. 4a**). Upon comparison, we observed that nearly half of the marker genes discovered by SIMBA overlap with the marker genes identified by Scanpy (**Supplementary Fig. 4a**). However, on inspection of the top non-overlapping marker genes, genes identified by SIMBA are found to be enriched only within certain groups of cells (**Supplementary Figs. 4b and 4c**) while genes identified by Scanpy but not by SIMBA include the housekeeping gene *B2M* and multiple ribosomal protein genes (e.g., *RPS3* and *RPS6*) that are expressed ubiquitously in all cell types (**Supplementary Figs. 4b and 4d**). Furthermore, a combination of different statistical tests proposed in Scanpy is required to recover the genes identified only by SIMBA. For example, IL7R[7] was identified only by using the t-test and FCER1A[8] was identified only by using the Wilcoxon rank-sum test, as also noted in the Scanpy's tutorial, while SIMBA successfully identified both *IL7R* and *FCER1A* as informative genes with a single procedure and without clustering the cells (**Fig. 2e and Supplementary Fig. 1b**). These examples illustrated some limitations of the clustering-based statistical-tests methods.

We also compared SIMBA with two recent clustering-free marker gene detection methods, Cell-ID[9] and singleCellHaystack[10]. Both Cell-ID and singleCellHaystack are applicable to only scRNA-seq data. Cell-ID identifies cell-specific marker genes to annotate cell types whereas singleCellHaystack identifies dataset-level marker genes. SIMBA consistently showed better cell type prediction accuracy than Cell-ID and higher cell type specificity than singleCellHaystack.

As Cell-ID identifies per-cell marker genes and singleCellHaystack identifies dataset-level marker genes, the two functionalities are compared with SIMBA's corresponding functionalities separately below.

Cell-ID identifies cell-specific marker gene signatures without a significance measure (by default, it selects the top 200 genes), then uses this list of genes to predict for each cell in the input dataset the most likely cell type based on a reference dataset. The identification of cell-specific marker genes corresponds to SIMBA's functionality of finding marker genes for each cell based on the proximity of their embeddings. To fairly compare the accuracy of the cell-specific markers identified by Cell-ID and SIMBA, we used the same procedure proposed by Cell-ID to predict cell types using the marker genes identified by each method.

To this end, we compared the cell type prediction accuracy of the two methods on the human pancreas scRNA-seq dataset which has been used also in the CellID's tutorial (https://rausellab.github.io/CelliD//vignettes/vign.html). To predict cell types, we compared the marker genes identified by Cell-ID or SIMBA against the reference marker genes of all human cell types provided by the Panglao database 11. This process is similar to the one described in the Cell-ID tutorial, but it takes into account the different numbers of marker genes per cell identified by each method. We note that although the cell type labels from the original study areis defined using clustering solutions derived by scRNA-seq, the prediction accuracy of predicting pancreas-specific cell types against all human reference cell types is a valid evaluation. In this comparison, SIMBA consistently shows better cell type prediction accuracy than CellID, especially when a small number of marker genes per cell is used (**Supplementary Fig. 6a-b**).

SingleCellHaystack identifies marker genes that have significantly different expression levels within a specific region of the cell embedding compared to the rest of the embedding. This functionality corresponds to the marker feature discovery using SIMBA metrics for cell type specificity.

Using the 10x PMBC scRNA-seq dataset, we've compared the cell type specificity of the marker gene sets identified by singleCellHayStack and SIMBA with different significance thresholds. As both methods do not use the cell type information to define the marker genes, we can use the cell type labels of the original study as the ground truth to evaluate the marker features' cell type specificity. The cell type specificity of the marker genes is calculated by the Gini index of the average log-normalized gene expression values per cell type. SIMBA consistently identified

marker genes with better cell type specificity compared to singleCellHaystack (**Supplementary Fig. 6c**).

Supplementary Note 4. Comparison of SIMBA in computational complexity

To make the run time and memory requirements of SIMBA clear, especially as compared to popular frameworks like the Python-based Scanpy and R-based Seurat, we applied all three methods against three datasets: the 10x PBMCs (3k) dataset, Pancreas RNA dataset[11], and the 10x 1.3M mouse neuron dataset. We also progressively down-sampled the 10x 1.3M mouse neuron dataset from the entire dataset to 500 cells.

Approach. In Supplementary Fig. 26, each plot shows the mean value of three iterations. Each analysis method was run four times on each dataset, the first iteration of each being discarded to yield N=3 iterations. Discarding the first iteration effectively mitigates inconsistencies related to caching that effect both the time and memory requirements of all methods. During application of SIMBA to the entire 1.3M cell dataset, restarting of the kernel was required regardless, thus only three iterations were performed. For both the time and memory results for each method, we grouped sub-routines into three general categories: preprocessing and I/O, embedding and dimension reduction, and feature discovery. We also denote separately with respect to SIMBA, the functions for graph generation and training. The sub-routines and their respective categorization included for each method are described in the table below. Plotting functions were excluded from this benchmark.

Datasets. For the 10x PBMC (3,000 cells) and Pancreas RNA dataset, we used the dataset accessible via the function: *simba.datasets.rna_10x_pbmc_3k()* and *simba.datasets.rna_pancreas()* functions, respectively. For the 10x mouse neuron dataset, we downloaded the .h5 file from the 10x website and progressively down-sampled at 500, 1,000, 2,500, 5,000, 10,000, 25,000, 50,000, 100,000, 250,000, 500,000, and 1,000,000 cells as well as the full dataset. Using default parameters, Seurat was only able to run up to 500,000 cells. Due to an inherent limitation of R, Seurat is unable to process large .h5 files, which prevented us from applying Seurat to the full dataset. On the 500 Gb machine used, the 1,000,000 cell down-sampling exceeded the memory available during the Seurat::FindAllClusters step.

Benchmarking methods. To benchmark time occupancy for methods that use Python, we wrote a decorator function that uses the built-in time package, which was then called with each sub-routine executed by SIMBA and Scanpy. To benchmark memory occupation for methods built in Python, we wrote a decorator function that uses both the psutil and tracemalloc libraries. Additionally, we wrote a script to capture the memory trace of the entire system, which serves as a ceiling on estimates for sub-routines that are passed to the system from python by PyTorch-BigGraph, which are unable to be traced by the previously described method. To monitor and benchmark run time in R, we used the Sys.time function between each Seurat function to record

a detailed execution time. To monitor and benchmark memory consumption in R, we used the gc library to calculate the memory allocated, before, during, and after each Seurat function executed. The memory shown in each figure represents the maximum memory required for a step within the functional category listed.

Machine. All analyses were run using a rented custom N1 Series Google Cloud instance configured with 24 vCPUs and 500 GB of RAM. The same virtual machine was used for all analyses in this comparison.

Results. For all datasets, Scanpy had lower run-time requirements than SIMBA. Run-times required by Seurat were slightly less than or similar to Scanpy. This is especially notable for the progressive down-sampling experiment wherein as the dataset size is increased beyond 25,000 cells, Seurat's run-time requirement ceases to increase and even reduces. Seurat is parameterizable such that trade-offs in memory and run-time enable appropriate scaling. Using the default parameters, the dataset was chunked such that the memory requirement continued to expand while the required run-time experienced a decrease with an increase in dataset size, up to 500,000 cells where Seurat was no longer able to run within the memory constraints of the machine (500 Gb). Progressive down-sampling of the large 1.3M mouse neuron dataset illustrates that Scanpy consistently required less memory than Seurat though the two methods scale at nearly the same rate upon dataset expansion (**Supplementary Fig. 26d**). In contrast, SIMBA has a much higher baseline memory requirement for even very small (N=500 cells) datasets though experiences a sub-linear trend in the maximum memory required upon dataset expansion. After 100,000 cells, SIMBA requires less memory to operate than Seurat and begins to approach the memory requirement trendline of Scanpy. The large maximum memory requirement by SIMBA for small datasets is largely attributable to the training procedure, which learns the embeddings of both cells and genes, as opposed to other tools that learns only cellular states. For a typical dataset, such as the 10x PBMCs (3,000 cells) dataset (**Supplementary Fig. 26a**) or the Pancreas dataset (**Supplementary Fig. 26b**), Seurat required nearly as much memory as SIMBA, while Scanpy required less than either. In any case, each method is reasonable to use on a machine with < 4Gb RAM (less than is currently standard for a common laptop computer).

Category	SIMBA	Scanpy	Seurat
I/O and preprocessing	simba.pp.filter_genes<br>simba.pp.cal_qc_rna<br>simba.pp.filter_cells_rna<br>simba.pp.normalize<br>simba.pp.log_transform<br>simba.pp.select_variable_genes<br>simba.tl.discretize<br>simba.tl.gen_graph (I/O time)	scanpy.pp.filter_cells<br>scanpy.pp.filter_genes<br>scanpy.pp.calculate_qc_metrics<br>scanpy.pp.normalize_total<br>scanpy.pp.log1p<br>scanpy.highly_variable_genes<br>scanpy.pp.regress_out	Seurat::CreateSeuratObject<br>Seurat::PercentageFeatureSet<br>Seurat::NormalizeData<br>Seurat::FindVariableFeatures<br>Seurat::ScaleData

		scanpy.pp.scale	
Graph Generation	simba.tl.gen_graph (I/O time excluded)		
Training	simba.tl.pbg_train		
Embedding	simba.read_embedding<br>simba.tl.umap (cells)<br>simba.tl.embed<br>simba.tl.umap (cells and genes)	sc.tl.pca<br>sc.pp.neighbors<br>sc.tl.umap	Seurat::RunPCA<br>Seurat::FindNeighbors<br>Seurat::UMAP
Gene Discovery	simba.tl.compare_entities	scanpy.tl.leiden<br>scanpy.tl.rank_genes_groups	Seurat::FindAllClusters<br>Seurat::FindAllMarkers

Supplementary Note 5: SIMBA embedding in scATAC-seq analysis

We investigated the peaks and k-mers that are embedded close to GATA1 (**Supplementary Fig. 8**). We observe that these peaks are open in all MEP cells and part of CMP cells, which are either mixed with or adjacent to MEP cells, and that these kmers are highly enriched in MEP cells and resemble the GATA1 TF motif.

To assess SIMBA's ability to cluster cell types based on scATAC-seq profiles, we compared SIMBA with specialized methods specifically designed for this task. We observed that SIMBA yields consistent embeddings of cells when using either a single feature (peaks) or multiple features (peaks and DNA sequences from within those peaks) as input to the graph. This comparison was performed across four scATAC-seq datasets of varying profiling technologies and organisms (**Supplementary Fig. 4**). Given these differences, to create a fair comparison we used the same set of features (i.e., peaks) for SIMBA as other methods. SIMBA's performance was compared against three of the top methods, including SnapATAC[12], *Cusanovich*2018[13], and cisTopic[14] recommended by our recent benchmark study[15]. This comparison was first made qualitatively based on UMAP visualization and then quantitatively based on clustering performance. SIMBA performed as well as or better than each of the methods evaluated. These results comparing SIMBA to scATAC-seq-specialized methods highlight SIMBA's wide utility for single-cell analyses (**Supplementary Fig. 5**).

We observe qualitatively in **Supplementary Fig. 9** that there were negligible differences in the resulting embedding generated by SIMBA, whether or not sequences were included as input features, in addition to peaks. To evaluate SIMBA performance using different features, in **Supplementary Fig. 10**, we applied a clustering evaluation framework that we developed previously to evaluate the goodness of the embeddings of cells generated by SIMBA[15]. Here, we supplement the qualitative observations shown in **Supplementary Fig. 9** with a quantitative comparison, applying this clustering evaluation framework to the embeddings generated by

SIMBA using (1) peaks or (2) peaks and sequences as the input. We applied SIMBA to three real scATAC-seq datasets: 10x PBMCs (5k cells), a human hematopoiesis (Buenrostro, 2018) with FACS-based cell type labels, and a sub-sampling of the sciATAC-seq mouse atlas and two synthetic bone marrow (“clean” and “noisy”) scATAC-seq datasets. We then apply three common clustering methods, hierarchical clustering, K-means clustering, and Louvain clustering to each resulting embedding and use ARI, AMI, and Homogeneity as metrics to evaluate the clustering quality, compared to the ground truth labels, for each cell. For all datasets and evaluation metrics, only minimal or inconsistent differences were observed, indicating that there is negligible impact on the embedding generated by SIMBA upon the inclusion of sequences as additional features. We speculate that there is inherent redundancy in the information contained by the peaks and their sequence content and that deeper investigation of feature contribution may illuminate advantages to featurization to be considered in future work.

Supplementary Note 6. Queries of marker features and cells in SIMBA space

We show the additional utility of the proximity queries in SIMBA embedding in the discovery of a subset of cells based solely on their proximity to a recovered marker feature by SIMBA and independent of any clustering solutions.

To demonstrate the point, we highlight that SIMBA discovered a master regulator Relb and its target genes *Nfkbie*, *Nfkbia*, and *Nfkb1* in the mouse skin SHARE-seq dataset (**Fig.4d, Supplementary Fig. 7**). Relb was not reported in the original study but literature supports that this hair-follicle-specific master regulator plays an important role during the hair follicle development[16-18]. Importantly, by querying the neighboring cells of Relb in the SIMBA embedding space, it is possible to identify the cell subpopulations with high activity of Relb. This helps refine the classification of the cell types based on the global clustering solution proposed by alternative methods.

We show that a distinct cell subpopulation with high Relb activity in both Relb expression and Relb motif accessibility can be identified based on the proximity of cells to Relb motifs and genes (**Supplementary Fig. 28a**). This subpopulation with high Relb activity mainly corresponds to the subpopulation of TAC-2 in the original annotation, revealing the potentially unreported heterogeneity within the defined cell type (**Supplementary Fig. 28c-d**). We further showed the coordinated regulation of Relb and its target genes within the subpopulation (**Supplementary Fig. 28 e-f**). This example clearly shows that SIMBA can uncover cell state from a master regulator without clustering and leverage the co-embedding of cells and features to discover a novel subset of cells with co-expressed gene modules. In principle, any marker feature with

high SIMBA cell type specificity scores can serve as a 'location of interest' in the SIMBA embedding space to query its proximal cells for further investigation by the users.

Supplementary Note 7: Comparison of SIMBA in batch correction

Multiple methods have now been developed to correct for the technical effects of sample preparation and data collection in single cells. To assess SIMBA's performance in removing batch effects, we compared it to Seurat3[19], LIGER[20] and Harmony[21], three top-performing batch correction methods recommended in a recent benchmark study[22].

Two datasets, including a mouse atlas dataset and a human pancreas dataset (see **Supplementary Table 2**), were used for the evaluation. The mouse atlas dataset is composed of two scRNA-seq subsets with shared cell types from different sequencing platforms. The human pancreas dataset is composed of five samples pooled from five distinct sources using four different sequencing techniques wherein not all cell types are shared across each sample.

To qualitatively compare these methods, we visualized cells of each dataset before and after batch-correction in UMAP plots (**Supplementary Fig. 19b,d**). To quantitatively evaluate the performance of each method, using the benchmarking pipeline laid out in Tran *et al*[22], we measured the conservation of biological information and batch effect removal based on three different metrics: average silhouette width (ASW), adjusted Rand index (ARI), and local inverse Simpson's index (LSI)[21] as in the previously-mentioned benchmark study[22] (**Supplementary Fig. 19a,c**). Each metric measures the relative mixing of class labels, where optimal performance is associated with maximal mixing in the batch labels and minimal mixing in the cell type labels.

Average Silhouette width (ASW)

Average Silhouette width is the mean value of Silhouette scores calculated from each cell. Silhouette width measures the relative closeness of cells with the same label compared to the cells with the different label and ranges from -1 to +1. Silhouette score for a data point with a label is calculated as

$$s(i) = \frac{b(i) - a(i)}{\max\{a(i), b(i)\}}$$

where $a(i)$ is the distance to the closest point with the same label, and $b(i)$ is the distance to the closest point with different labels. A high Silhouette score means the point is located more closely with the same label, where a low Silhouette score closer to -1 means the point is located closer with different labels than that of itself. The ideal batch correction result will give a low ASW score for batch labels as the point is well mixed with other batches and a high ASW score for the cell type labels as the cells of the

same cell type should cluster together after the batch correction. The final score is calculated as the median ASW scores from 20 subsets of randomly sampled 80% cells.

Average Rand Index (ARI)

To evaluate the cell type purity, the true cell type labels and the k-means clustering solution were used to calculate the cell type ARI. To evaluate the batch correction performance, the true batch labels and the k-means clustering solution were used to calculate the batch ARI. The final ARI was calculated as the median ARI scores of 20 subsets comprised of randomly sampled 80% cells for batches and cell types, respectively. A superior batch correction will have a high cell type ARI (high agreement between the clustering solution and the true cell type labels), and a low batch ARI (the clustering solution is not mainly driven by batches and clusters contain cells with well-mixed batch labels).

Local Inverse Simpson's Index (LISI)

Local Inverse Simpson's Index (LISI) [21] measures the local batch and cell type mixing. For each data point, it considers the Gaussian kernel weighted distribution of labels in its neighborhood with a perplexity argument. We set perplexity to 50 40 as in the previous benchmark study. Using the weighted neighborhood label distribution, the inverse Simpson's index is calculated as $\frac{1}{\sum_l p(l)}$ where l is the batch or cell type labels and $p(l)$ is the probability of each label in the local neighborhood obtained with the kernel. For each cell, the LISI is the expected number of cells to be sampled locally before a cell of the same label is sampled. A perfect batch correction will have a cell type LISI (cLISI) of 1 and a batch LISI (integration LISI, iLISI) close to the number of batches. The final LISI score was calculated as the average LISI scores of all cells.

The "Raw" batch correction results are the first 50 principal components of the horizontally concatenated gene-by-cell expression count matrix using *stats::prcomp* in R package with centering and scaling. The "Raw, preprocessed" batch correction used the preprocessed data with log normalization with scaling factor 10^4 and selection of 3000 highly variable genes with Seurat v3 with no restriction on the minimum number of cells and genes.

For batch correction using Seurat v3, default options are used for pancreas dataset whereas for mouse atlas dataset no cutoff was used for the minimum number of cells and genes as in Tran *et al*.[22]. The dimension of the batch corrected embedding is set as 50 dimensions following the default option for *Seurat::RunPCA* and for the consistency with SIMBA.

For batch correction using LIGER, the same arguments are used (lambda = 5, nrep = 3) are used for *liger::optimizeALS* in Tran *et al*. other than the number of factors k was set as 50 for consistency with other methods for both datasets.

For batch correction using Harmony, the same arguments are used as in Tran *et al*.[22] other than the number of dimensions of the output embedding was set to 50 instead of 20. We note that the output embedding of 20 dimensions would result in the similar result as when used 50 dimensions in these methods.

Supplementary Note 8: **Comparison of SIMBA in multi-omics integration**

Seurat3 and LIGER are two of the most widely-adopted methods for single-cell data integration. Here, we demonstrate that SIMBA outperforms these methods on two separate datasets, the recently published SHARE-seq mouse skin dataset and the similarly recent 10x PBMCs multiome dataset (**Supplementary Table 2**). We focus on Seurat3 and LIGER as they have explicit documentation for the task of integrating scRNA-seq and scATAC-seq data.

We first qualitatively evaluated these methods by inspecting UMAP visualization plots. For the SHARE-seq dataset, we observed that all three methods perform comparably well in mixing cells of two modalities though LIGER generated particularly small and noisy clusters (**Supplementary Fig. 23b)**. For the 10X PBMCs dataset, SIMBA resulted in the best mixing of cells belonging to each modality whereas other methods clustered cells separately within the originating modalities (**Supplementary Fig. 23d**). We next quantitatively assessed the integration performance of each method using four metrics that measure the distances between matched cells in the integrated space. In addition to the commonly-used metrics including anchoring distance, Silhouette index, and Fraction in the same cluster, we developed an additional metric, *anchoring distance rank* (ADR)*,* which represents the normalized rank of the distance between matching cells. If two matching cells from scRNA-seq and scATAC-seq are mutually closest to one another, their ADR will be close to 0 and thus a minimized ADR is ideal. Overall SIMBA showed the best performance according to ADR as well as cluster agreement while showing comparable or better performance according to the remaining metrics for both datasets (**Supplementary Fig. 23a,c**).

Anchoring distance

The Anchoring distance was proposed in Dou et al., 2020[23] and is the normalized distance between the matched cells of two modalities (e.g. RNA and ATAC). Here we considered the Euclidean distance and normalized the distance by the mean of the distances calculated between random pairs of cells. The number of pairs randomly sampled was set to 10% of the total number of cells.

Anchoring distance rank

Given that the anchoring distance does not account for the local density of cells, we propose a new metric entitled *anchoring distance rank* (ADR). The ADR is based on the normalized rank of the distance between the matched cells of two modalities. For each cell x_{ij} with cell identity i and modality j, the distance between the cell and all the other cells of the other modality j', $d\left(x_{ij}, x_{kj'}\right), k = 1, \dots, N$ is calculated, where N is the total

number of cells. Then the rank of $r_i = d(x_{ij}, x_{ij'})$ within the calculated distances is normalized by the number of pairs $N-1$ to obtain the final anchoring rank $m_i = \frac{r_i - 1}{N-1}$. For each cell, an anchoring rank of 0 indicates an ideal modality integration performance as the matched cells are closest to each other in the embedding.

Silhouette index
The silhouette index was calculated as described in 10) based on the cluster assignment wherein each cluster consists of two cells, one cell from a scRNA-seq dataset and one cell from a scATAC-seq dataset.

Fraction in the same cluster
Fraction in the same cluster was calculated as the fraction of the matched cells from two modalities in the same cluster. The clusters of cells were generated using Louvain algorithm and the number of clusters is equal to the number of cell types in the dataset.

The modality integration procedure for Seurat v3 and LIGER follows the tutorial provided by the authors (Seurat v3: https://satijalab.org/seurat/archive/v3.1/atacseq_integration_vignette.html; LIGER: http://htmlpreview.github.io/?https://github.com/welch-lab/liger/blob/master/vignettes/Integrating_scRNA_and_scATAC_data.html).

Both Seurat v3 and LIGER formulate the modality integration task between scRNA-seq and scATAC-seq data as a batch correction task between scRNA-seq and gene activity matrix constructed from scATAC-seq. In Seurat v3, the gene activity score of a gene is calculated as the sum of the read counts in the peaks that falls within from 2kb upstream of the TSS to the end of the gene body. In LIGER, this score is calculated as the sum of all read counts that falls within 3kb upstream of the TSS to the end of the gene body.

The “Raw” results start from a scRNA-seq count matrix and a gene activity matrix calculated by Seurat v3. Filtering for the shared genes in both modalities resulted in 16738 genes for the SHARE-seq mouse skin dataset and 11045 genes for the 10X PBMCs dataset. Gene-by-cell gene expression matrix and gene activity matrix were horizontally concatenated along matching rows (genes). The output embedding is the first 20 principal components calculated by the R function *stats::prcomp* with centering and scaling.

For the modality integration using Seurat v3, the gene expression count was filtered using the default parameters *min.cells = 3* and *min.features = 200*. The co-embedding was created as described in the tutorial of the package using the scRNA-seq. The output embedding consists of the first 50 principal components, which is the default option of *Seurat::RunPCA*.

For the modality integration using LIGER, the gene expression count and gene activity matrices were normalized and filtered for the genes that are shared between both matrices. The values

were then scaled according to the tutorial. In applying LIGER to the SHARE-seq mouse skin dataset, the function, *liger::optimizeALS* was used with the default parameters, k = 20 and lambda = 5. The scRNA-seq dataset was indicated as the reference in the function, *liger::quantile_norm* as described in the documentation. The scRNA-seq and scATAC-seq modalities of the 10X PBMC multiome dataset were unable to be aligned using the default parameters. Thus *lambda = 30* and *max.iters = 100* were used for the *liger::optimizeALS* function and the scATAC-seq dataset was indicated as the reference using the *liger::quantile_norm* function to ensure a better alignment.

Supplementary Note 9: Parameter tuning in SIMBA

We have performed a grid search to select the optimal hyperparameters and use the same hyperparameters for all the experiments across 15 datasets. The only parameter that needs to be tuned during the training is the "weight decay". However, this can be empirically optimized based on the training sample size (i.e., the total number of edges) of the dataset, and therefore we provide an automatic procedure to determine the optimal weight decay parameter. "*si.tl.pbg_train(auto_wd=True)*". All the experiments across 5 tasks in 15 datasets were performed with this procedure.

In addition, we also provide several training metrics (they can be plotted using the function "*si.pl.pbg_metrics()*") to monitor the training process so the users can further fine-tune the auto-generated weight decay parameter based on these training metrics if needed.

References:

1. Cao, Z.J. & Gao, G. Multi-omics single-cell data integration and regulatory inference with graph-linked embedding. *Nat Biotechnol* (2022).
2. Bilous, M. et al. Metacells untangle large and complex single-cell transcriptome networks. *BMC Bioinformatics* **23**, 336 (2022).
3. Zhao, Y., Cai, H., Zhang, Z., Tang, J. & Li, Y. Learning interpretable cellular and gene signature embeddings from single-cell transcriptomic data. *Nat Commun* **12**, 5261 (2021).
4. Tayyebi, Z., Pine, A.R. & Leslie, C.S. Scalable sequence-informed embedding of single-cell ATAC-seq data with CellSpace. *bioRxiv 2022.05.02.490310* (2022).
5. Wu, L.Y. et al. Starspace: Embed all the things! *Thirty-Second AAAI Conference on Artificial Intelligence* (2018).
6. Lynch, A.W. et al. MIRA: joint regulatory modeling of multimodal expression and chromatin accessibility in single cells. *Nat Methods* **19**, 1097-1108 (2022).
7. Lawson, B.R. et al. Interleukin-7 is required for CD4+ T cell activation and autoimmune neuroinflammation. *Clinical immunology* **161**, 260-269 (2015).
8. Shin, J.-S. & Greer, A.M. The role of FcεRI expressed in dendritic cells and monocytes. *Cellular and Molecular Life Sciences* **72**, 2349-2360 (2015).

9. Cortal, A., Martignetti, L., Six, E. & Rausell, A. Gene signature extraction and cell identity recognition at the single-cell level with Cell-ID. *Nat Biotechnol* (2021).
10. Vandenbon, A. & Diez, D. A clustering-independent method for finding differentially expressed genes in single-cell transcriptome data. *Nat Commun* **11**, 4318 (2020).
11. Baron, M. et al. A single-cell transcriptomic map of the human and mouse pancreas reveals inter-and intra-cell population structure. *Cell systems* **3**, 346-360. e344 (2016).
12. Fang, R. et al. Comprehensive analysis of single cell ATAC-seq data with SnapATAC. *Nat Commun* **12**, 1337 (2021).
13. Cusanovich, D.A. et al. The cis-regulatory dynamics of embryonic development at single-cell resolution. *Nature* **555**, 538-542 (2018).
14. Bravo Gonzalez-Blas, C. et al. cisTopic: cis-regulatory topic modeling on single-cell ATAC-seq data. *Nat Methods* **16**, 397-400 (2019).
15. Chen, H. et al. Assessment of computational methods for the analysis of single-cell ATAC-seq data. *Genome Biology* **20**, 241 (2019).
16. Bellet, M.M., Zocchi, L. & Sassone-Corsi, P. The RelB subunit of NFκB acts as a negative regulator of circadian gene expression. *Cell cycle* **11**, 3304-3311 (2012).
17. Krieger, K. et al. NF-κB participates in mouse hair cycle control and plays distinct roles in the various pelage hair follicle types. *Journal of investigative dermatology* **138**, 256-264 (2018).
18. Gugasyan, R. et al. The transcription factors c-rel and RelA control epidermal development and homeostasis in embryonic and adult skin via distinct mechanisms. *Molecular and cellular biology* **24**, 5733-5745 (2004).
19. Stuart, T. et al. Comprehensive Integration of Single-Cell Data. *Cell* **177**, 1888-1902 e1821 (2019).
20. Welch, J.D. et al. Single-Cell Multi-omic Integration Compares and Contrasts Features of Brain Cell Identity. *Cell* **177**, 1873-1887 e1817 (2019).
21. Korsunsky, I. et al. Fast, sensitive and accurate integration of single-cell data with Harmony. *Nat Methods* **16**, 1289-1296 (2019).
22. Tran, H.T.N. et al. A benchmark of batch-effect correction methods for single-cell RNA sequencing data. *Genome Biol* **21**, 12 (2020).
23. Dou, J. et al. Unbiased integration of single cell multi-omics data. *bioRxiv*, 2020.2012.2011.422014 (2020).

Supplementary Figures

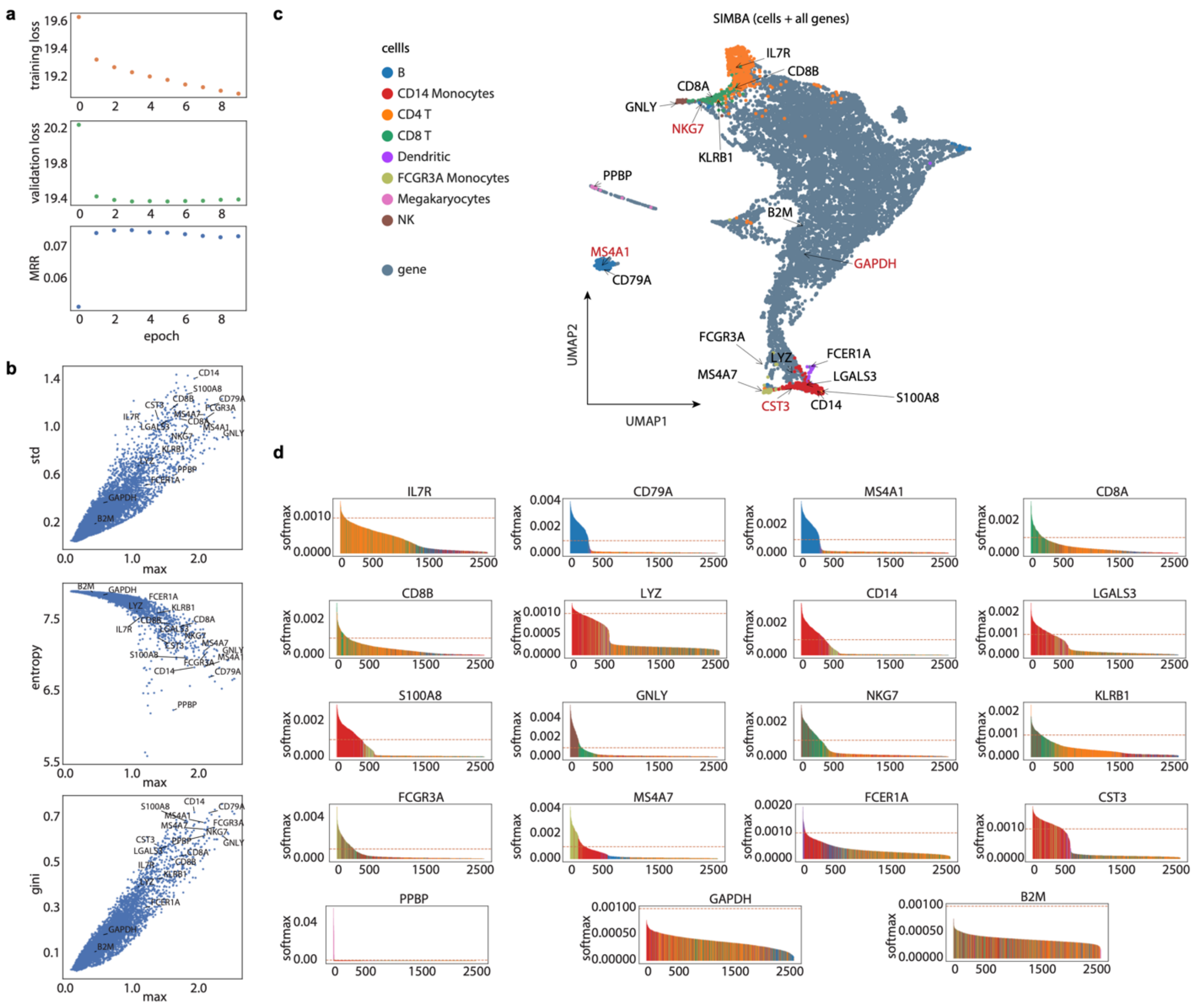

Supplementary Figure 1. SIMBA analysis of the scRNA-seq 10x PBMCs dataset.

- **a.** Three default metrics used to evaluate SIMBA training procedure, including training loss (top), validation loss (middle), mean reciprocal rank (MRR)
- **b.** SIMBA metric plots of genes. All the genes are plotted according to the Gini index against max score, standard deviation (std) against max score, and entropy against max score, respectively. The same set of genes as in Figure 2c are highlighted. Red dotted line shows the FDR 0.1 threshold.
- **c.** UMAP visualization of the SIMBA embeddings of cells and all genes. Genes highlighted in (b) are also highlighted in the UMAP plot.
- **d.** SIMBA barcode plots of the genes highlighted in (b).

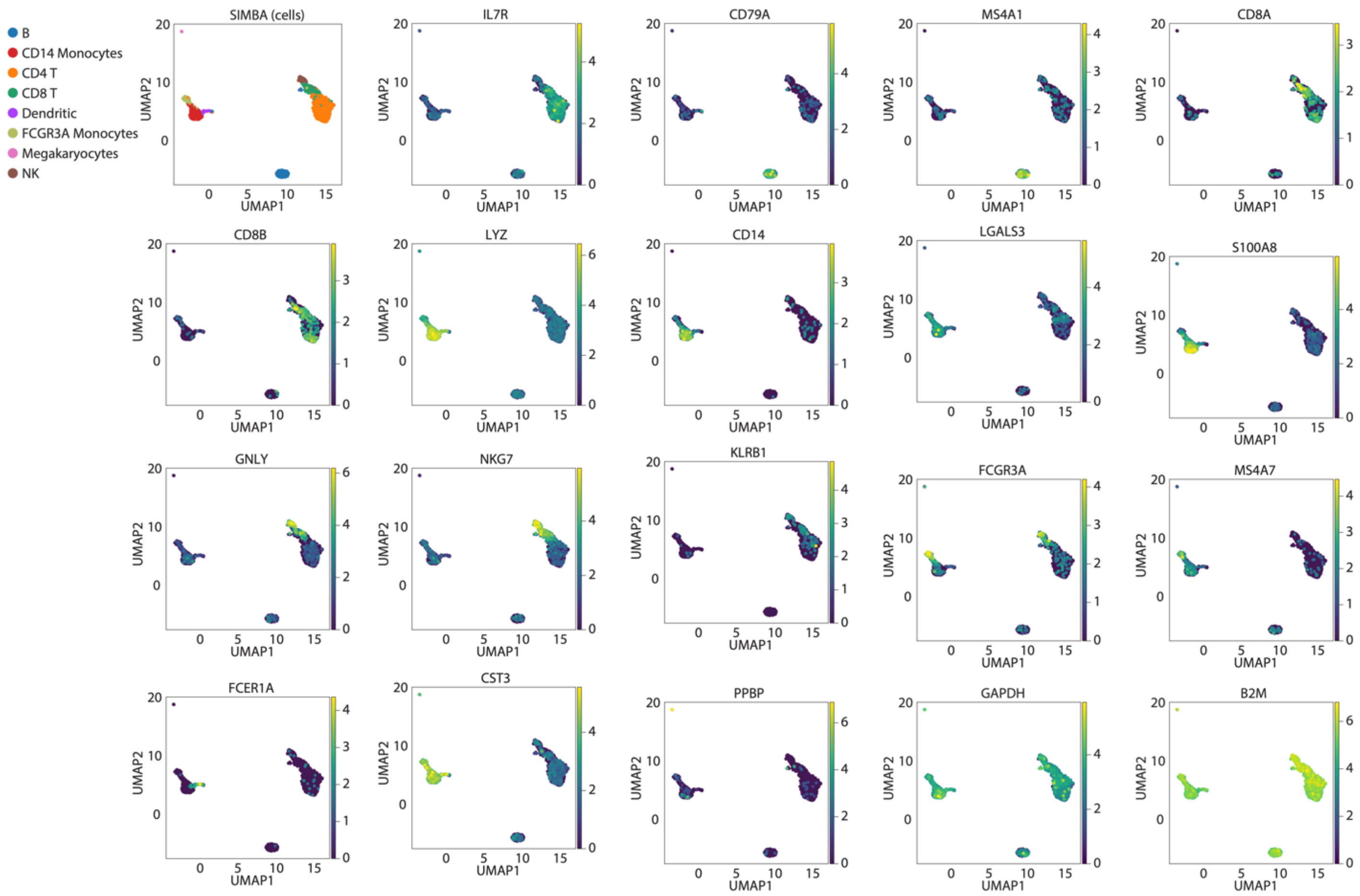

Supplementary Figure 2. UMAP visualization of the SIMBA embeddings of cells in the scRNA-seq 10x PBMCs dataset. Cells are colored by cell type labels and gene expression of the genes highlighted in Figure 2c.

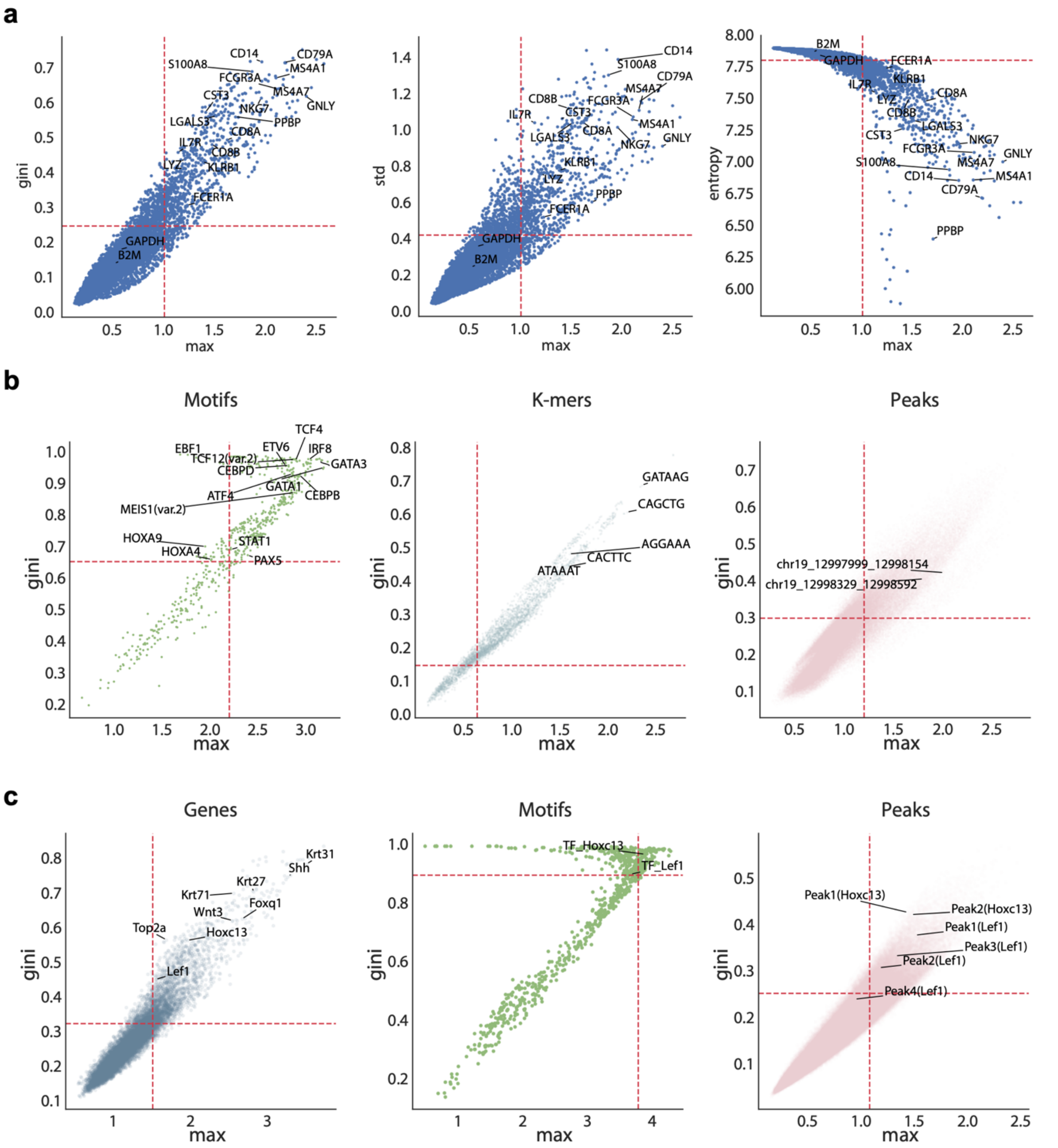

Supplementary Figure 3. SIMBA metric plots with a significance threshold

a. SIMBA metric plots for the scRNA-seq dataset as in Supplementary Figure 1 with the FDR threshold 0.1.
b. SIMBA metric plots for the scATAC-seq dataset as in Figure. 3d with the FDR threshold 0.1.
c. SIMBA metric plots for the SHARE-seq dataset as in Figure. 4b with the FDR threshold 0.1.

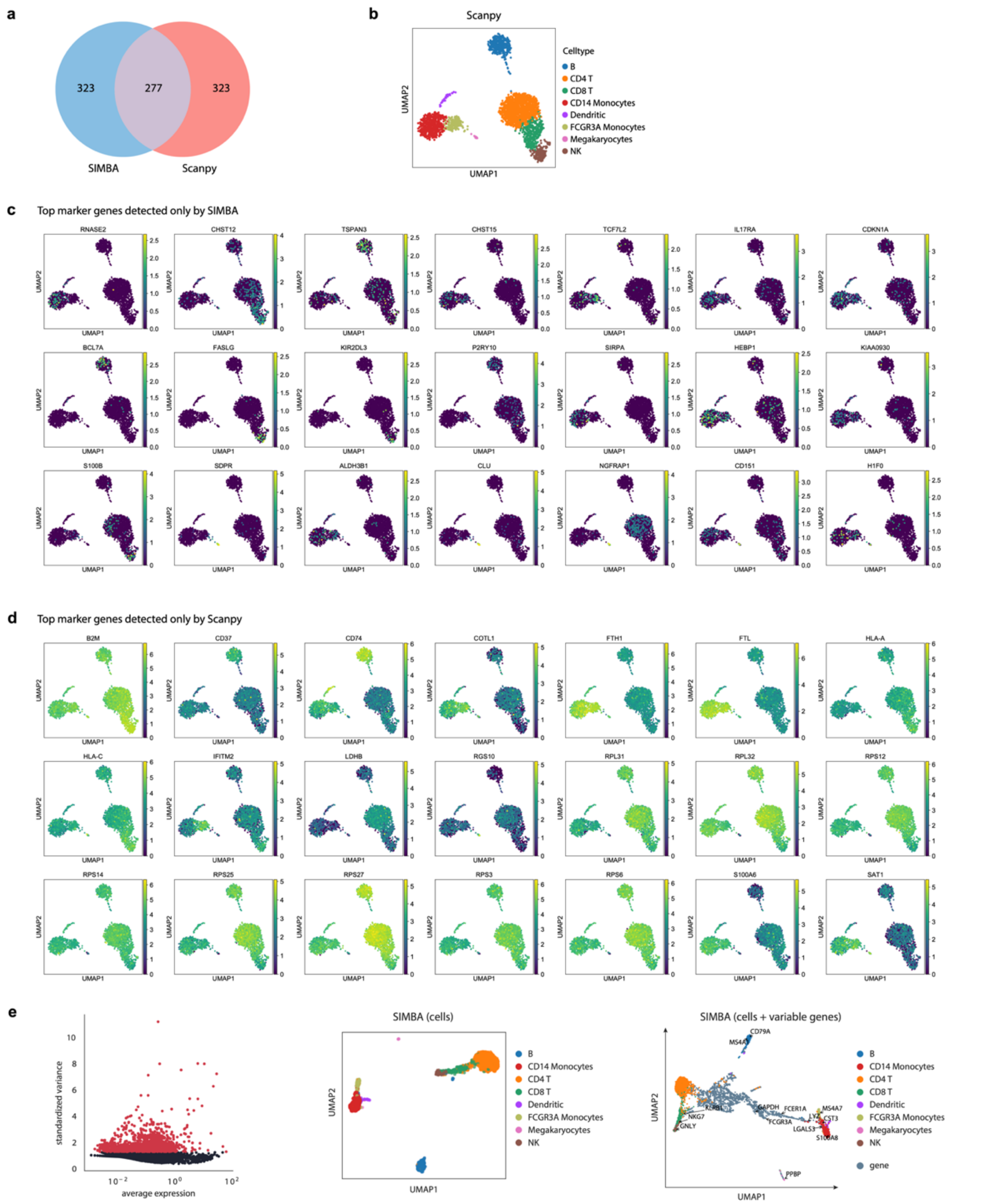

Supplementary Figure 4. Comparison of SIMBA with Scanpy on the scRNA-seq 10x PBMCs dataset.

- **a.** Venn diagram of top marker genes identified by SIMBA and Scanpy
- **b.** Scanpy-derived UMAP visualization of cells colored by cell type
- **c.** Top marker genes detected only by SIMBA. Colored by intensity of gene expression.
- **d.** Top marker genes detected only by Scanpy. Colored by intensity of gene expression.
- **e.** SIMBA embedding result after implementing variable gene selection. Left: variable gene selection step implemented in SIMBA. Middle: UMAP visualization of SIMBA embeddings of cells. Right: UMAP visualization of SIMBA embeddings of cells and variable genes.

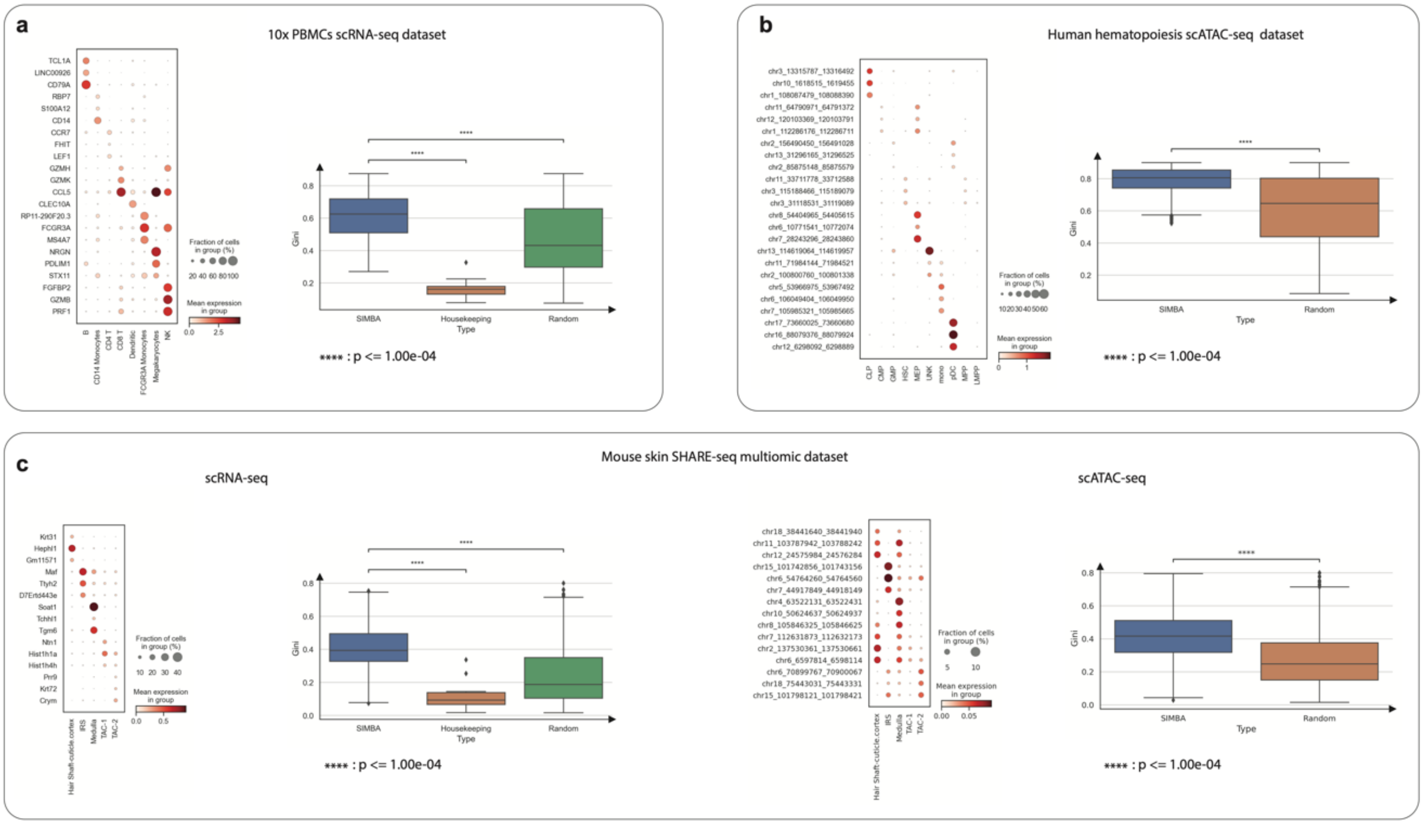

Supplementary Figure 5. Quantitative validation of marker features identified by SIMBA (***: 1.00e-04 < p <= 1.00e-03, ****: p <= 1.00e-04, ns: not significant, two-sided Mann-Whitney-Wilcoxon test). For scRNA-seq datasets, left: dotplot of gene expression for top SIMBA marker genes across cell types; right: boxplots of distribution of Gini index scores for SIMBA marker genes, house-keeping genes, and 500 randomly selected genes. For scATAC-seq datasets, left: dotplot of chromatin accessibility for top SIMBA marker peaks across cell types; boxplots of distribution of Gini index scores for SIMBA marker peaks, and 5,000 randomly selected peaks. a) Markers genes on the 10x PBMCs dataset (p values are 8.489e-15 and 8.701e-30). b) Marker peaks on the human hematopoiesis scATAC-seq dataset (p value is 0.0e+00). c) Marker genes and marker peaks on the mouse skin SHARE-seq multiomic dataset (scRNA-seq: p values are 1.306e-11 and 3.388e-50; scATAC-seq: p value is 0.0e+00). Boxes in the boxplots indicate first quarter, median, and third quarter of the data. The whiskers extend to ±1.5 x the inter-quartile range (IQR).

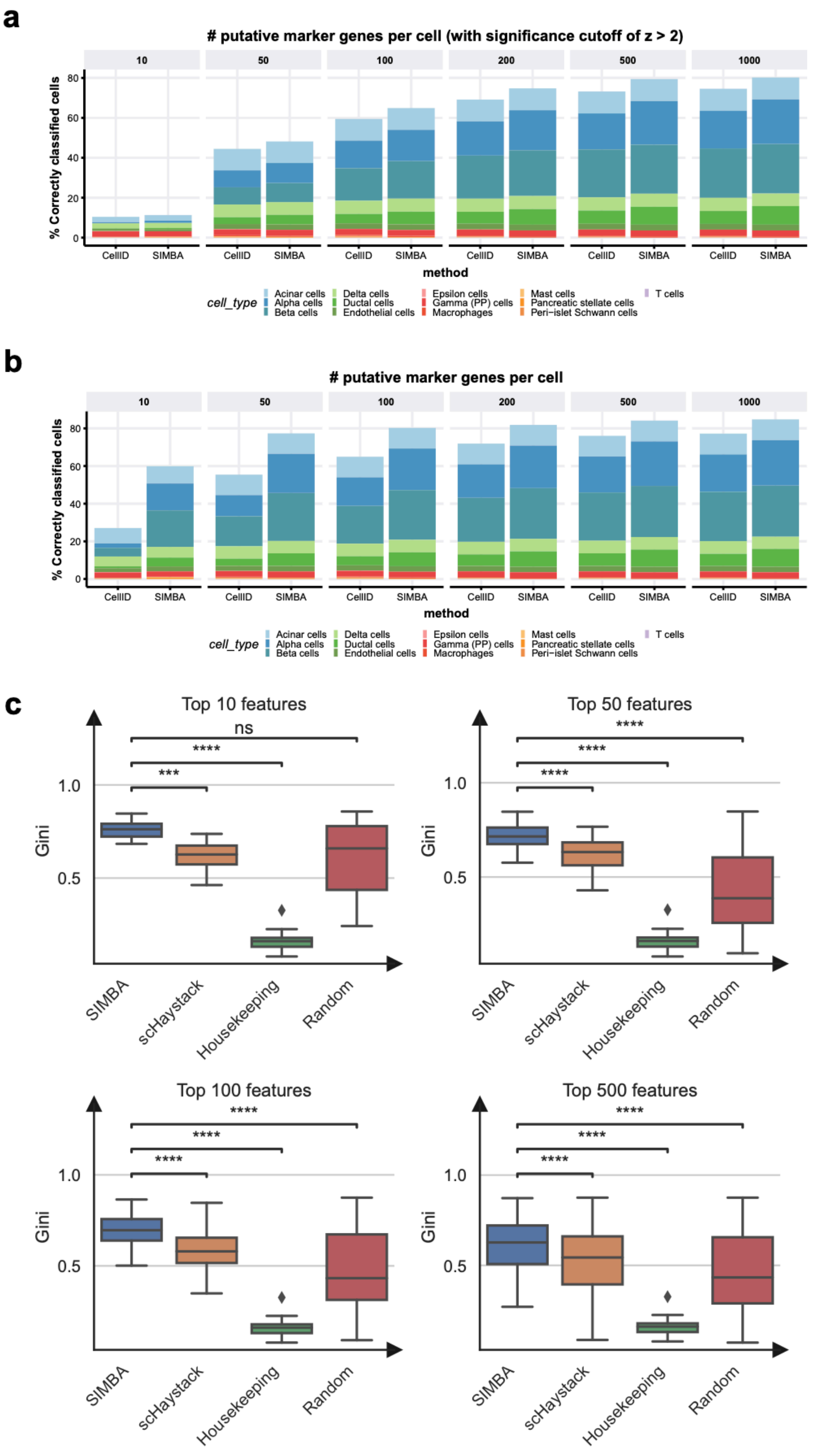

Supplementary Figure 6. Comparison of SIMBA with clustering-free marker gene detection methods.

- **a.** Classification accuracy of cell type prediction based on cell-type-specific marker genes identified by Cell-ID and SIMBA with a significance cutoff (z >2).
- **b.** Classification accuracy of cell type prediction based on cell-type-specific marker genes identified by Cell-ID and SIMBA without a significance cutoff.

c. Cell-type-specificity of the marker genes identified by singleCellHaystack and SIMBA. Gini index scores of the top 10, 50, 100 and 500 features are plotted in each panel (***: 1.00e-04 < p <= 1.00e-03, ****: p <= 1.00e-04, ns: not significant, two-sided Mann-Whitney-Wilcoxon test). For top 10 features: p values are 7.68e-04, 1.01e-5, 0.141, respectively; for top 50 features: p values are 1.52e-07, 3.89e-11, 5.98e-12, respectively; for top 100 features: p values are <1e-13, <1e-13, 1.41e-12, respectively; for top 500 features: p values are all <1e-13. Boxes in the boxplots indicate first quarter, median, and third quarter of the data. The whiskers extend to $\pm$1.5 x the inter-quartile range (IQR).

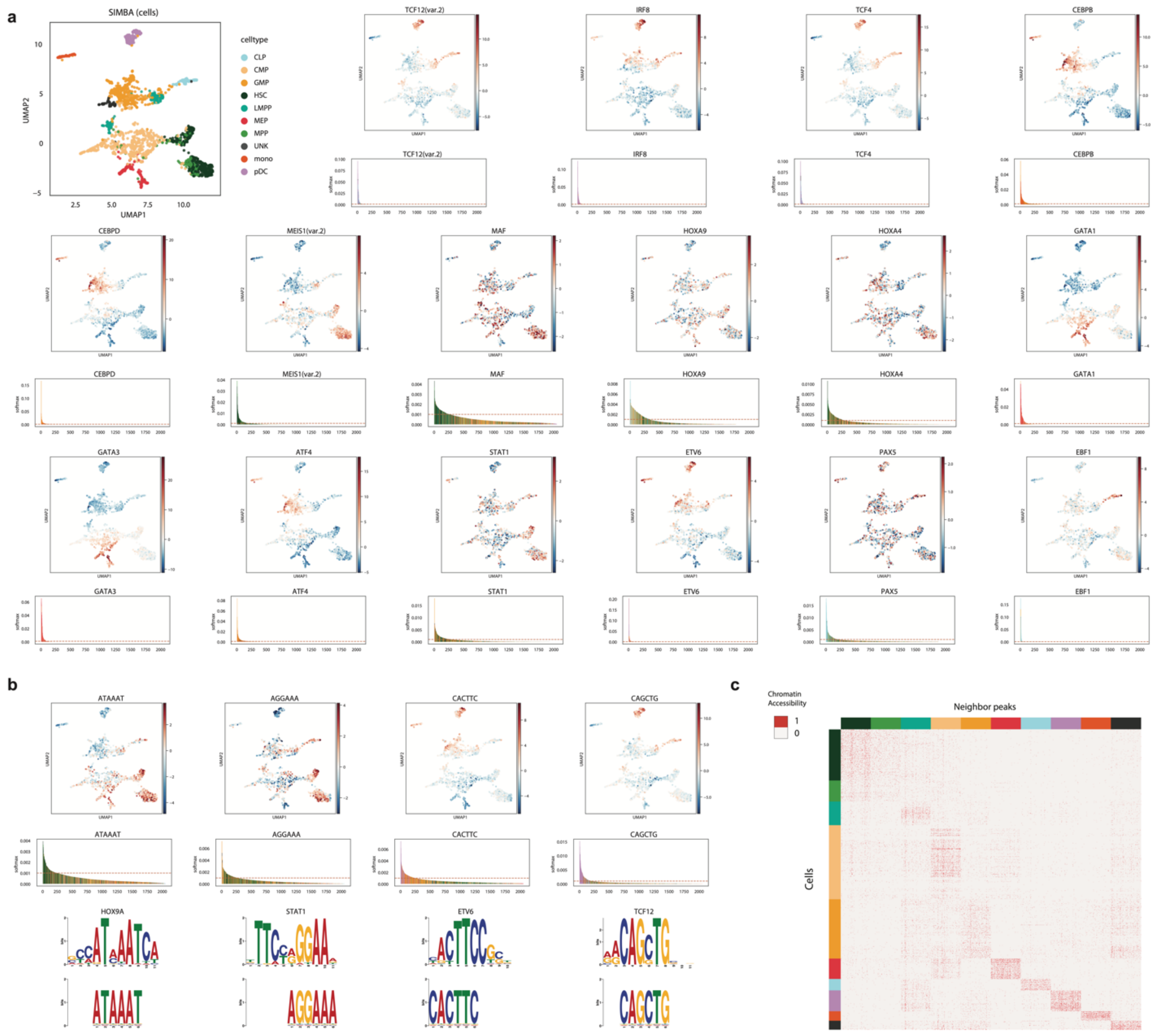

Supplementary Figure 7. SIMBA analysis of the *Buenrostro2018* dataset

- **a.** UMAP visualization of SIMBA embeddings of cells colored by cell type (top-left), and TF activity scores of TF motifs calculated with chromVAR, respectively. The SIMBA barcode plot of each TF motif is shown below the UMAP plot.
- **b.** Top: UMAP visualization of SIMBA embeddings of cells colored by TF activity scores of k-mers calculated with chromVAR. Middle: SIMBA barcode plots of the corresponding k-mers. Bottom: the matching known motif against the enriched k-mer sequences.
- **c.** Heatmap of cells against neighboring peaks of each cell type that are selected in the SIMBA co-embedding space. Chromatin accessibility is binary and colored accordingly.

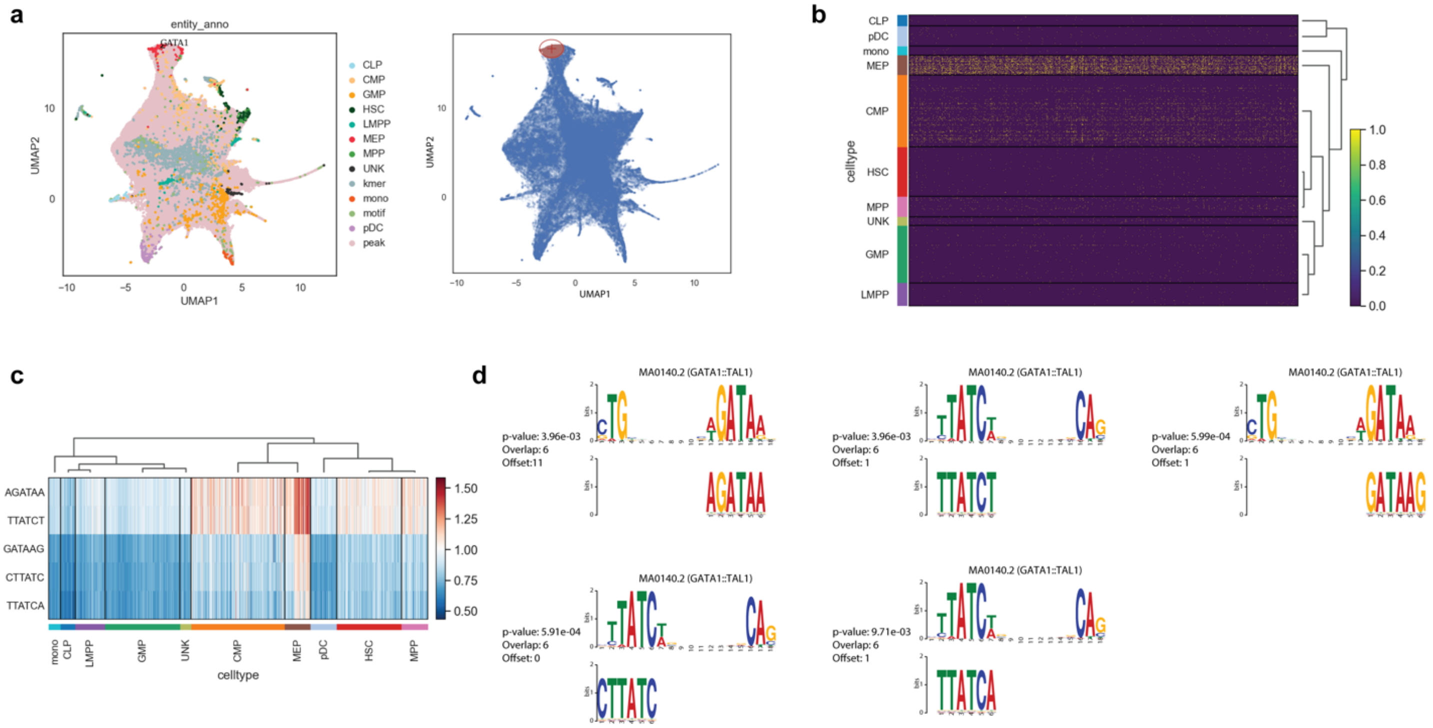

Supplementary Figure 8. Evaluation of the TF motif GATA1 and its neighboring entities. a) Left: UMAP visualization of SIMBA embeddings of cells and features including TF motifs, k-mers, and peaks. The TF motif GATA1 is indicated. Right: UMAP visualization of SIMBA embeddings of cells and features. The neighboring region of GATA1 is indicated. b) Heatmaps of chromatin accessibility for peaks in the neighboring region (columns) across all cell types (rows) for the human hematopoiesis dataset. c) Heatmaps of normalized read counts for *k*-mers in the neighboring region (rows) across all cell types (columns) for the human hematopoiesis dataset. (d) k-mers in the neighboring region (bottom) and their resembling TF GATA1 motif (top) sequence logos (Tomtom statistical measure of motif-motif similarity).

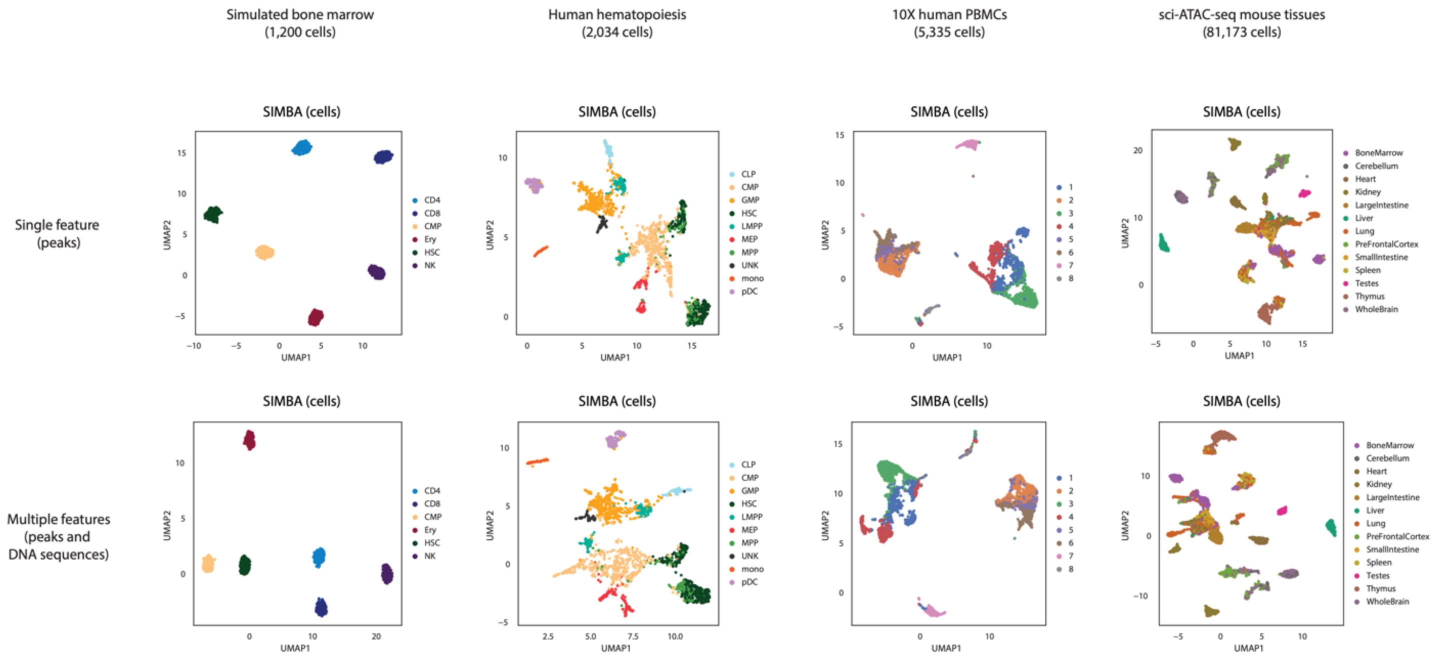

Supplementary Figure 9. Comparison of SIMBA performance using scATAC-seq peaks and DNA sequence content vs only scATAC-seq peaks. **Top**: UMAP visualization of SIMBA embeddings of cells for each indicated dataset generated from only scATAC-seq peak information. **Bottom**: UMAP visualization of SIMBA embeddings of cells for each indicated dataset generated from scATAC-seq peak information and DNA sequence content information.

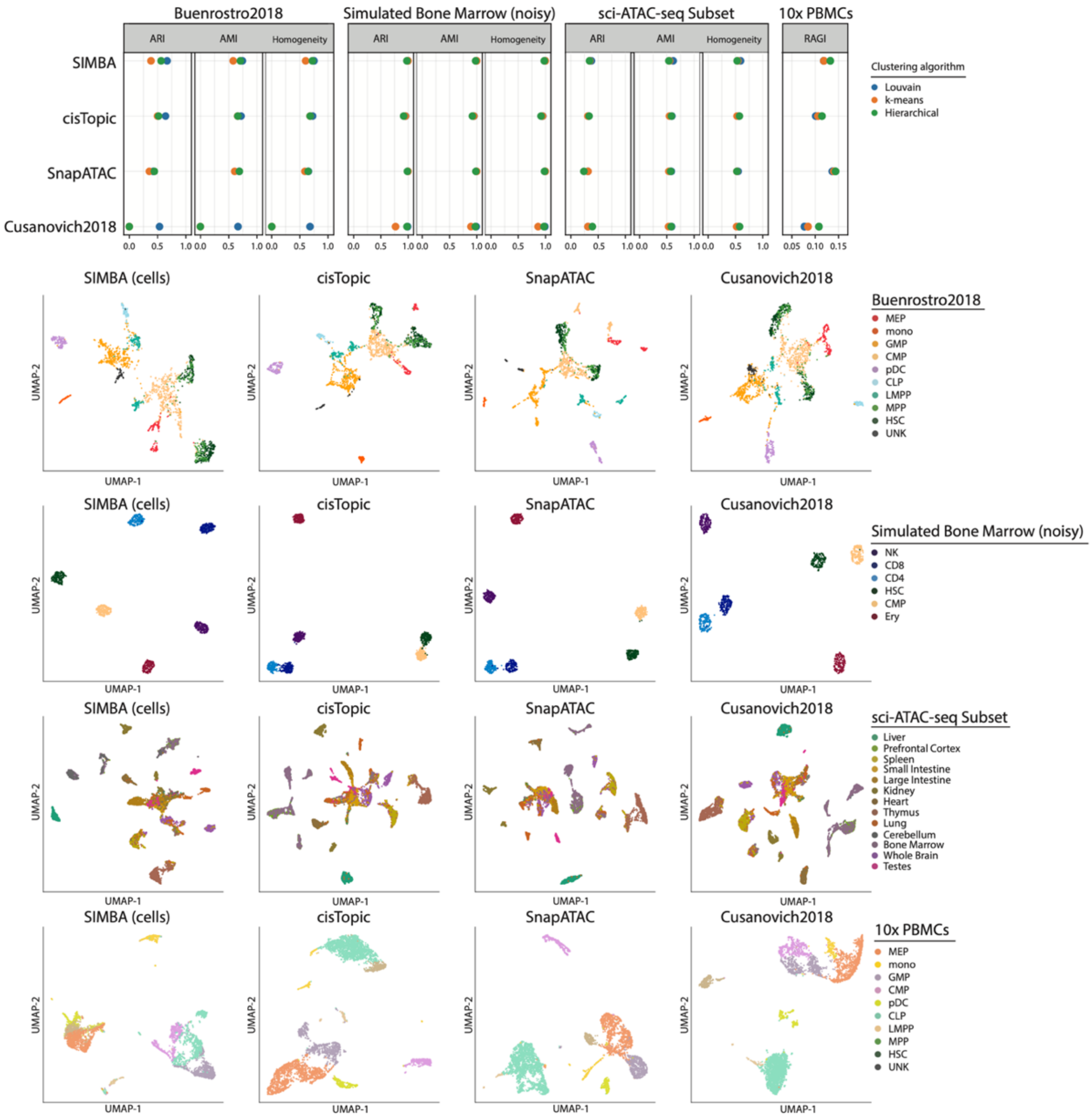

Supplementary Figure 10. Benchmark of SIMBA against top-performing scATAC-seq analysis methods.

Top: Evaluation of SIMBA and other methods including cisTopic, SnapATAC, *Cusanovich2018* for scATAC-seq analysis using metrics 1) ARI, AMI, and Homogeneity for datasets with ground truth cell type labels and 2) Residual Average Gini Index (RAGI) for the 10x PBMCs dataset without ground truth labels.
Bottom: UMAP visualization of feature matrices produced by each method on each dataset colored by cell type annotation or cluster label.

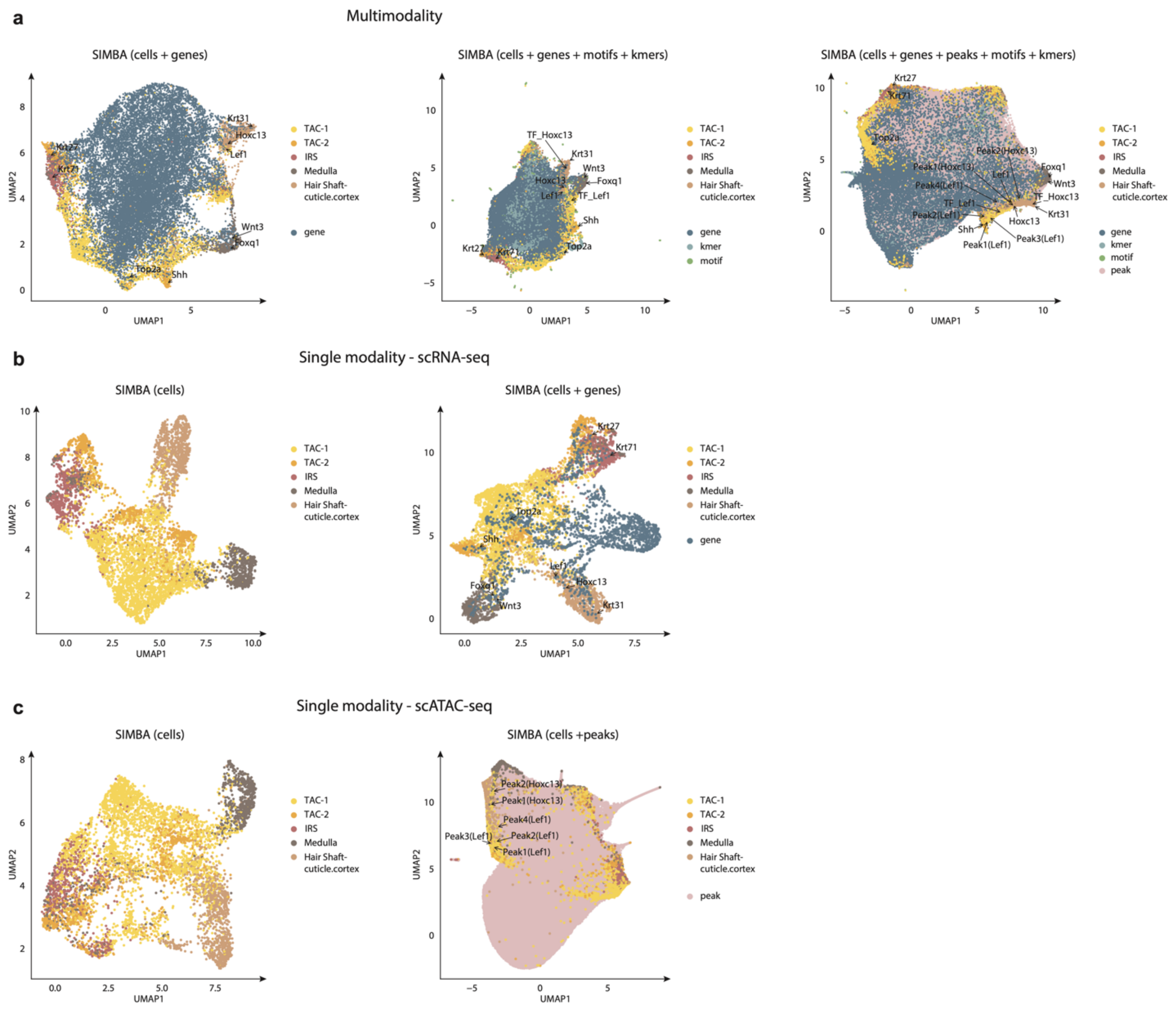

Supplementary Figure 11. SIMBA multimodal analysis of the SHARE-seq hair follicle dataset.

- **a.** SIMBA embedding results when both gene expression and chromatin accessibility are encoded in the graph. Left: UMAP visualization of SIMBA embeddings of cells and genes. Middle: UMAP visualization of SIMBA embeddings of cells along with genes, TF motifs, and k-mers. Right: UMAP visualization of SIMBA embeddings of cells along with genes, peaks, TF motifs, and k-mers.
- **b.** SIMBA embedding results when only gene expression is encoded in the graph. Left: UMAP visualization of SIMBA embeddings of cells. Right: UMAP visualization of SIMBA embeddings of cells and variable genes.
- **c.** SIMBA embedding results when only chromatin accessibility is encoded in the graph. Left: UMAP visualization of SIMBA embeddings of cells. Right: UMAP visualization of SIMBA embeddings of cells and peaks.

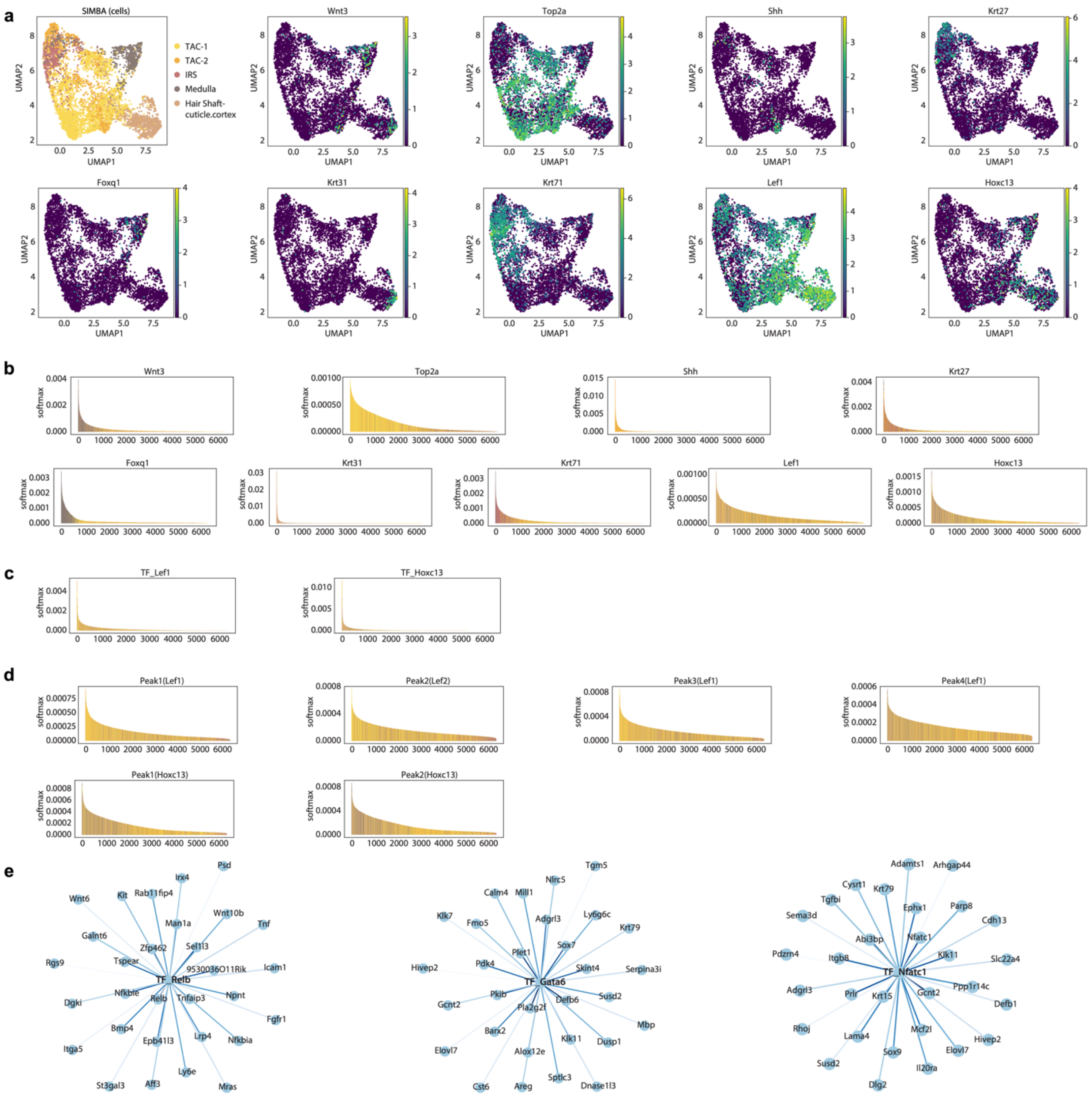

Supplementary Figure 12. Cell type specific marker genes and the target genes of master regulators identified by SIMBA in the SHARE-seq hair follicle subset dataset.

- **a.** UMAP visualization of SIMBA embeddings of cells colored by cell type and gene expression intensity.
- **b.** SIMBA barcode plots of each gene plotted above.
- **c.** SIMBA barcode plots of TF motifs *Lef1* and *Hoxc13.*
- **d.** SIMBA barcode plots of peaks near the loci of *Lef1* and *Hoxc13.*
- **e.** Top 30 target genes of the master regulators *Relb, Gata6,* and *Nfatc1* as inferred by SIMBA.

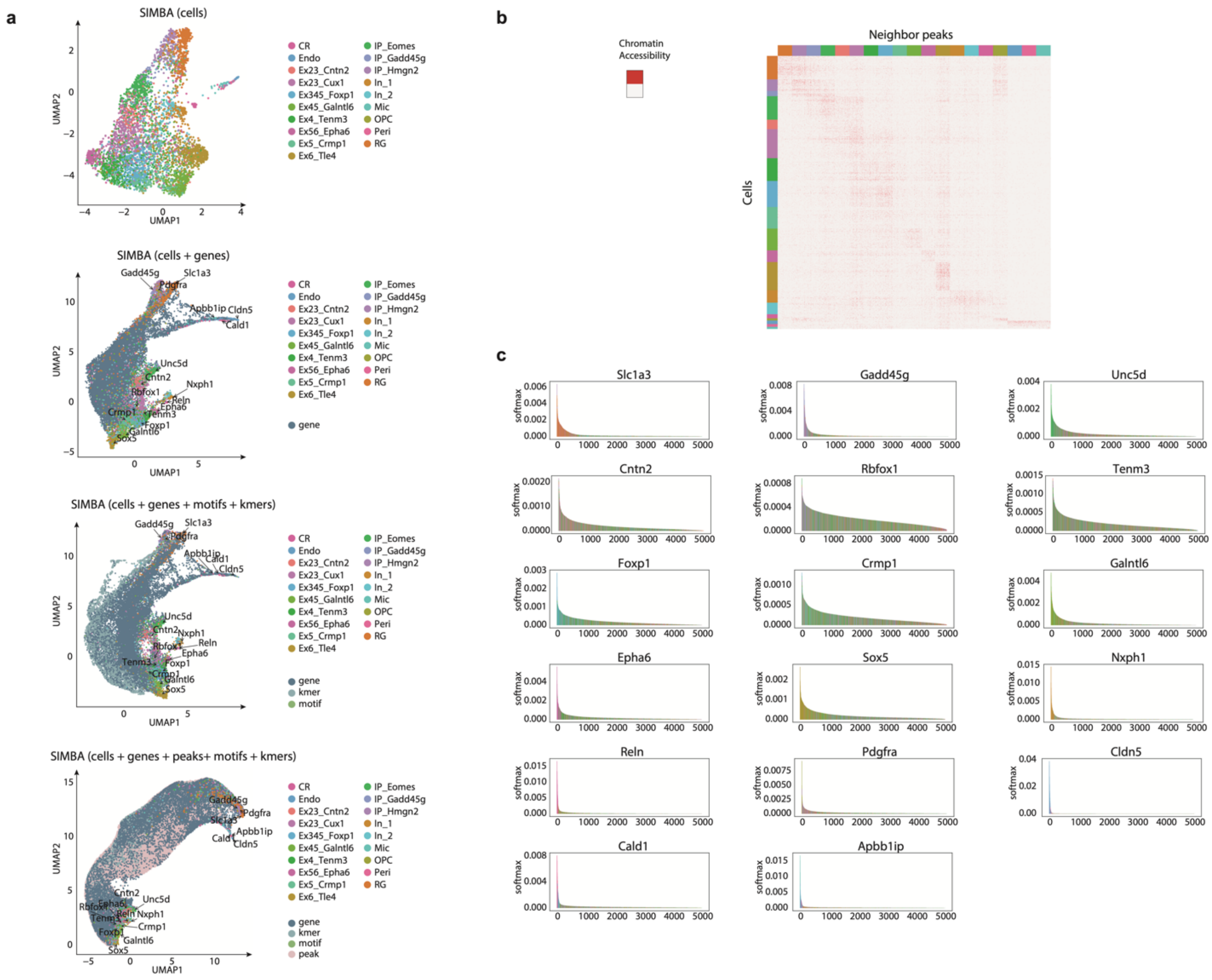

Supplementary Figure 13. SIMBA multimodal analysis of the SNARE-seq mouse cerebral cortex dataset.

- **a.** From top to bottom: UMAP visualization of SIMBA embeddings of (i) cells (ii) genes alongside cells (iii) genes, motifs, and k-mers alongside cells (iv) genes, peaks, motifs, and k-mers alongside cells.
- **b.** Heatmap of cells against neighboring peaks of each cell type that are selected in the SIMBA co-embedding space. Chromatin accessibility is binary and colored accordingly.
- **c.** SIMBA barcode plots of the genes highlighted in (a).

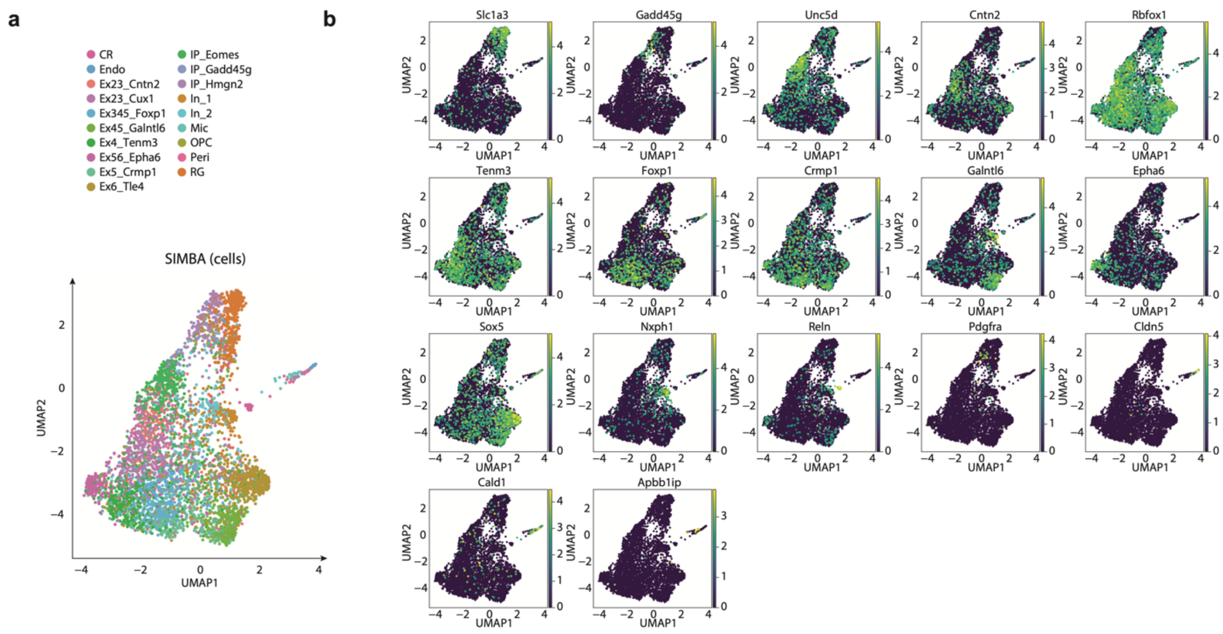

Supplementary Figure 14. UMAP visualization of SIMBA embeddings of cells in the SNARE-seq mouse cerebral cortex dataset. Cells are colored by cell type labels (a) and gene expression (b) of the genes highlighted in **Supplementary Figure 13**.

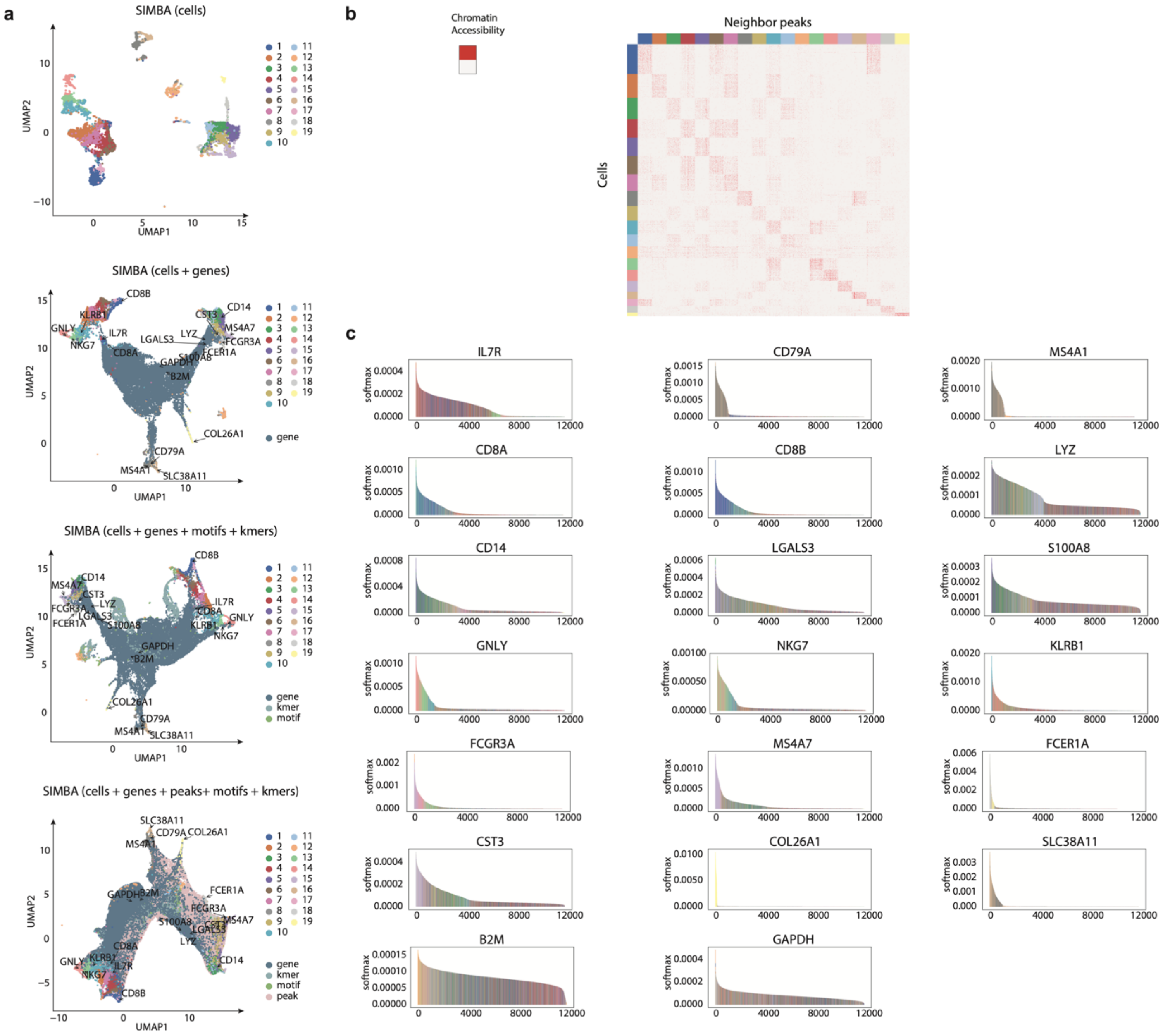

Supplementary Figure 15. SIMBA multimodal analysis of the 10x multiome PBMCs dataset.

- **a.** From top to bottom: UMAP visualization of SIMBA embeddings of (i) cells (ii) genes alongside cells (iii) genes, motifs, and k-mers alongside cells (iv) genes, peaks, motifs, and k-mers alongside cells.
- **b.** Heatmap of cells against neighboring peaks of each cluster that are selected in the SIMBA co-embedding space. Chromatin accessibility is binary and colored accordingly.
- **c.** SIMBA barcode plots of the genes highlighted in (a).

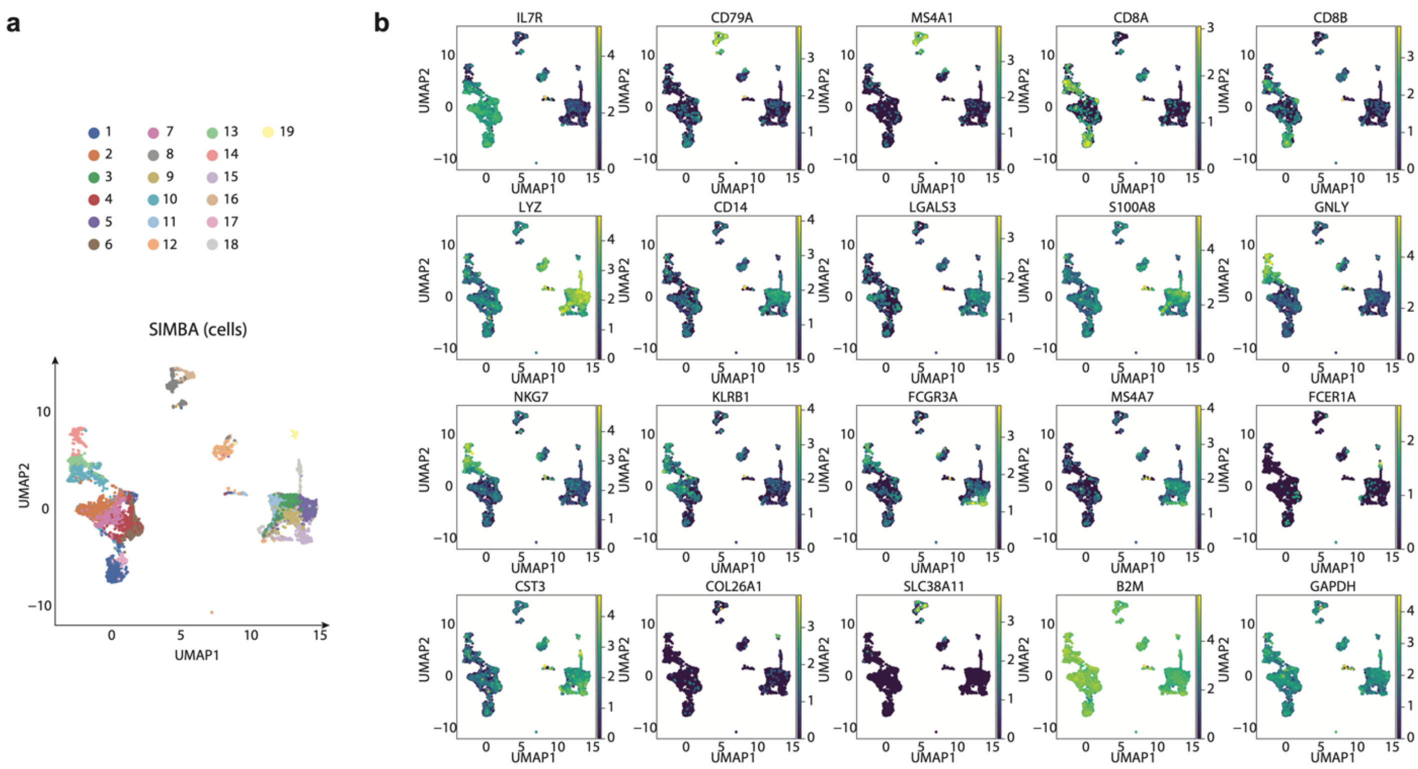

Supplementary Figure 16. UMAP visualization of SIMBA embeddings of cells in the 10x multiome PBMCs dataset. Cells are colored by cluster labels (a) and gene expression (b) of the genes highlighted in **Supplementary Figure 15**.

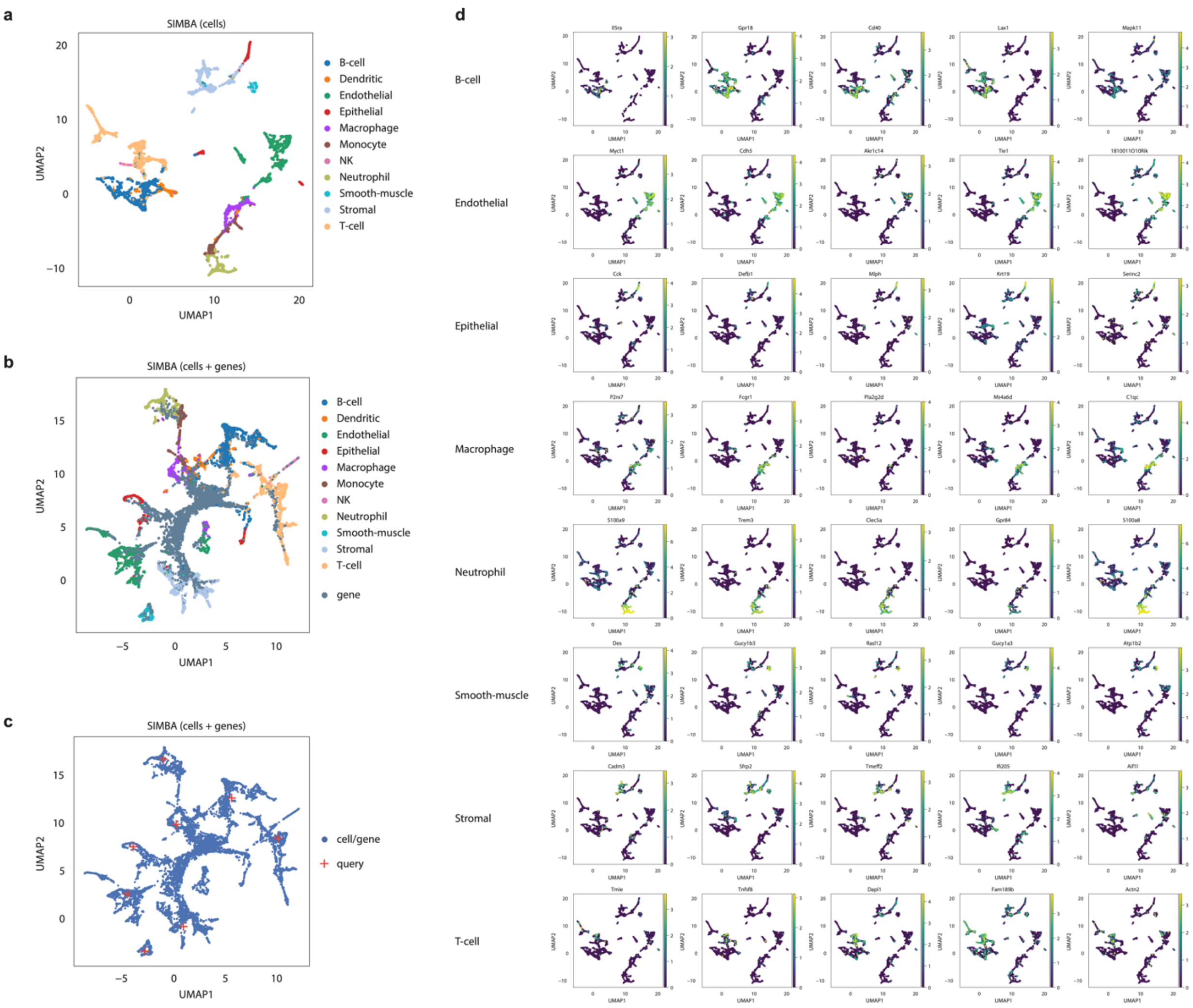

Supplementary Figure 17. SIMBA-inferred marker genes for the scRNA-seq mouse atlas dataset in batch correction analysis.

- **a.** UMAP visualization of SIMBA embeddings of cells colored by cell type.
- **b.** UMAP visualization of SIMBA embeddings of cells and genes.
- **c.** UMAP visualization of SIMBA embeddings of cells and genes. Biological “query” points are highlighted with a red “+”. Nearby informative genes are colored accordingly.
- **d.** UMAP visualization of SIMBA embeddings of cells colored by indicated gene expression intensity, separated by cell type.

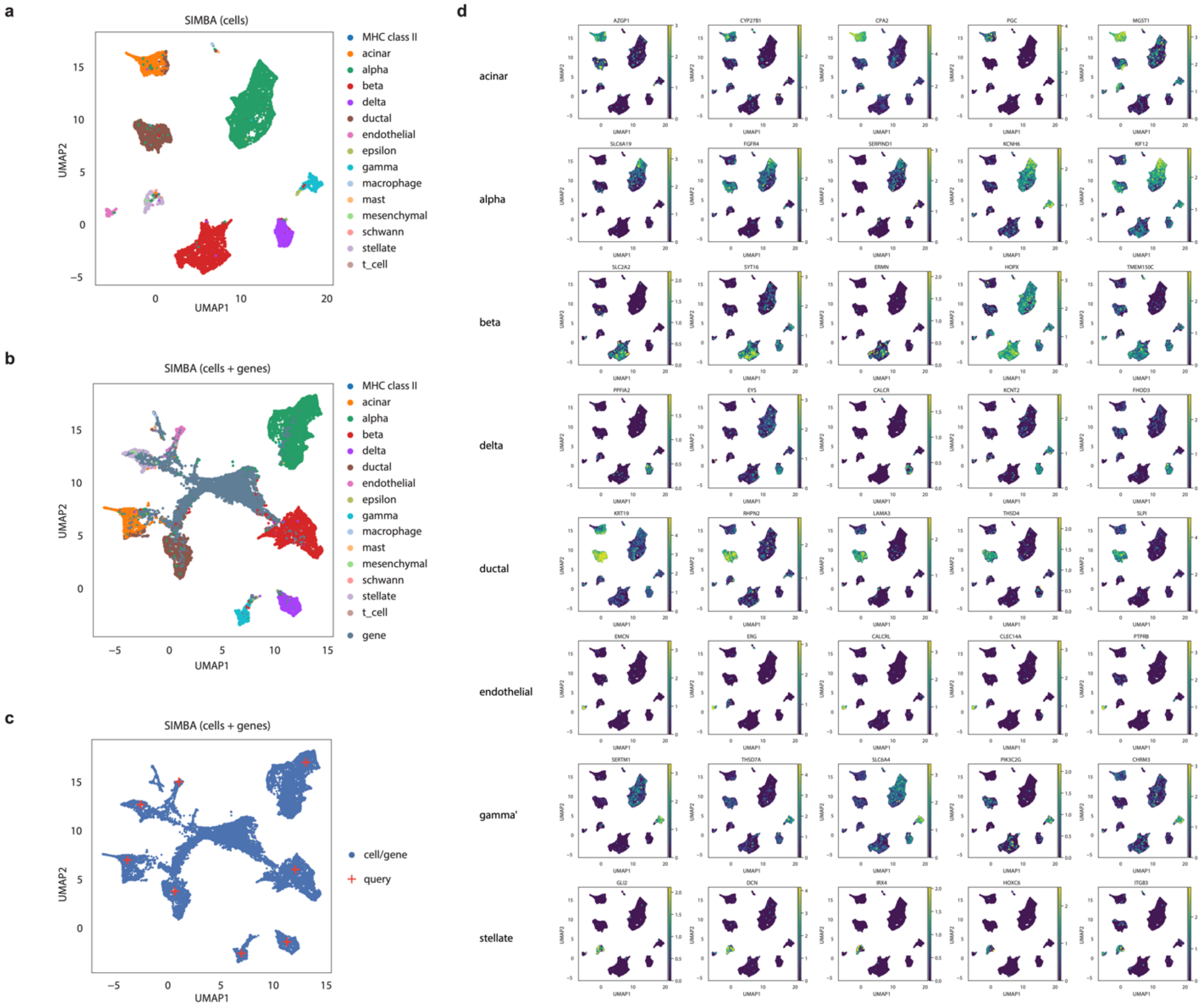

Supplementary Figure 18. SIMBA-inferred marker genes for the scRNA-seq human pancreas dataset in batch correction analysis.

- **a.** UMAP visualization of SIMBA embeddings of cells colored by cell type.
- **b.** UMAP visualization of SIMBA embeddings of cells and genes.
- **c.** UMAP visualization of SIMBA embeddings of cells and genes. Biological "query" points are highlighted with a red "+". Nearby informative genes are colored accordingly.
- **d.** UMAP visualization of SIMBA embeddings of cells colored by indicated gene expression intensity, separated by cell type.

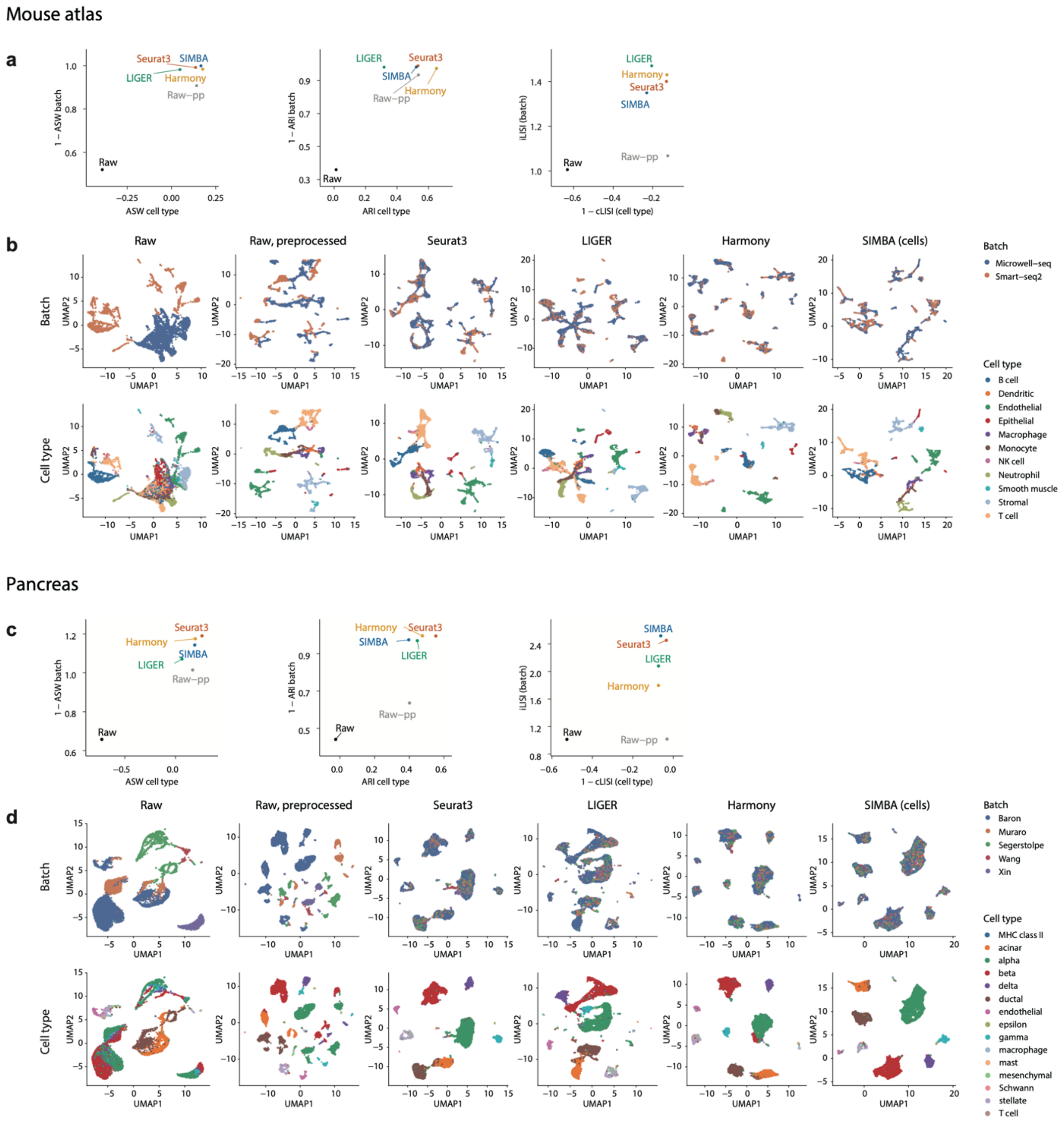

Supplementary Figure 19. Comparison of SIMBA to other methods for batch correction of the mouse atlas (a-b) and human pancreas scRNA-seq datasets (c-d).

a, c. Quantitative comparison of SIMBA with three other batch correction methods including Seurat3, LIGER and Harmony, using, left-to-right: average silhouette width (ASW), adjusted Rand index (ARI), and local inverse Simpson's index (LISI)

b, d. UMAP visualization of raw and preprocessed data alongside the batch corrected results produced by Seurat3, LIGER, Harmony, and SIMBA. Colored by technology (top) and cell type (bottom).

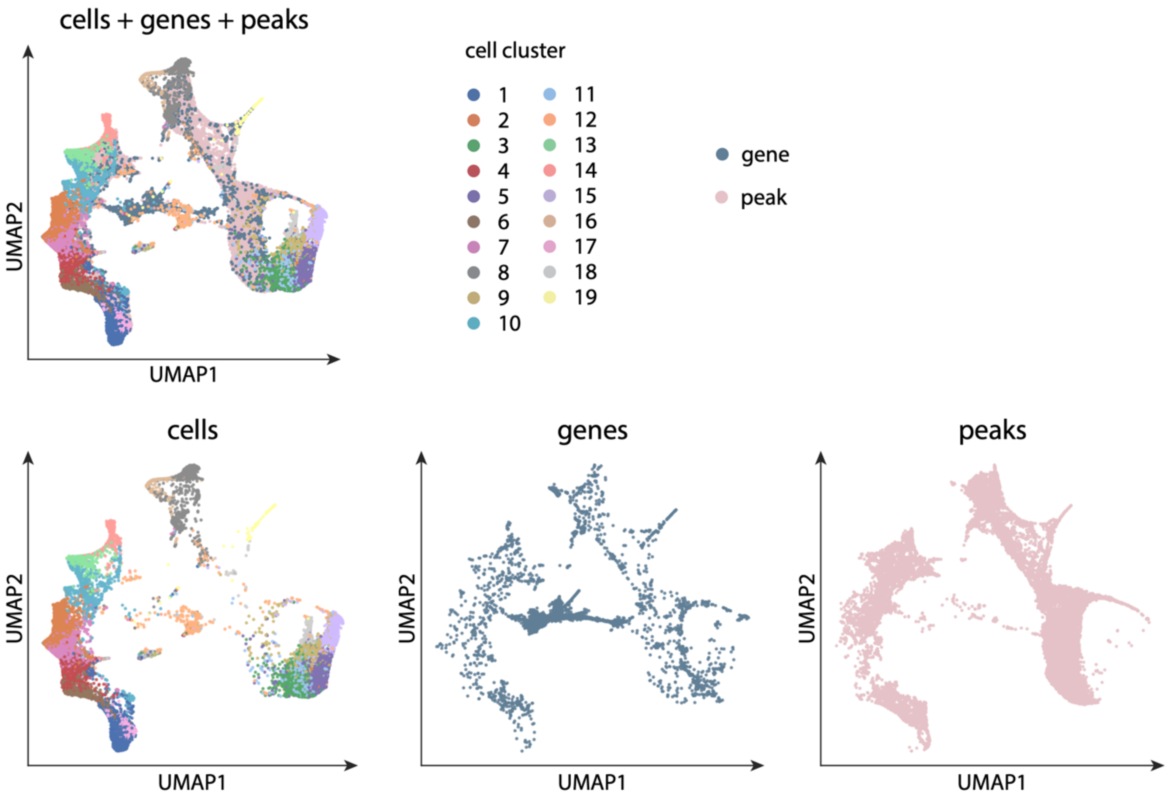

Supplementary Figure 20. Top: UMAP visualization of SIMBA embeddings of cells, genes, and peaks with two cell modalities integrated. Bottom: SIMBA embeddings of cells, genes, and peaks visualized separately in UMAP.

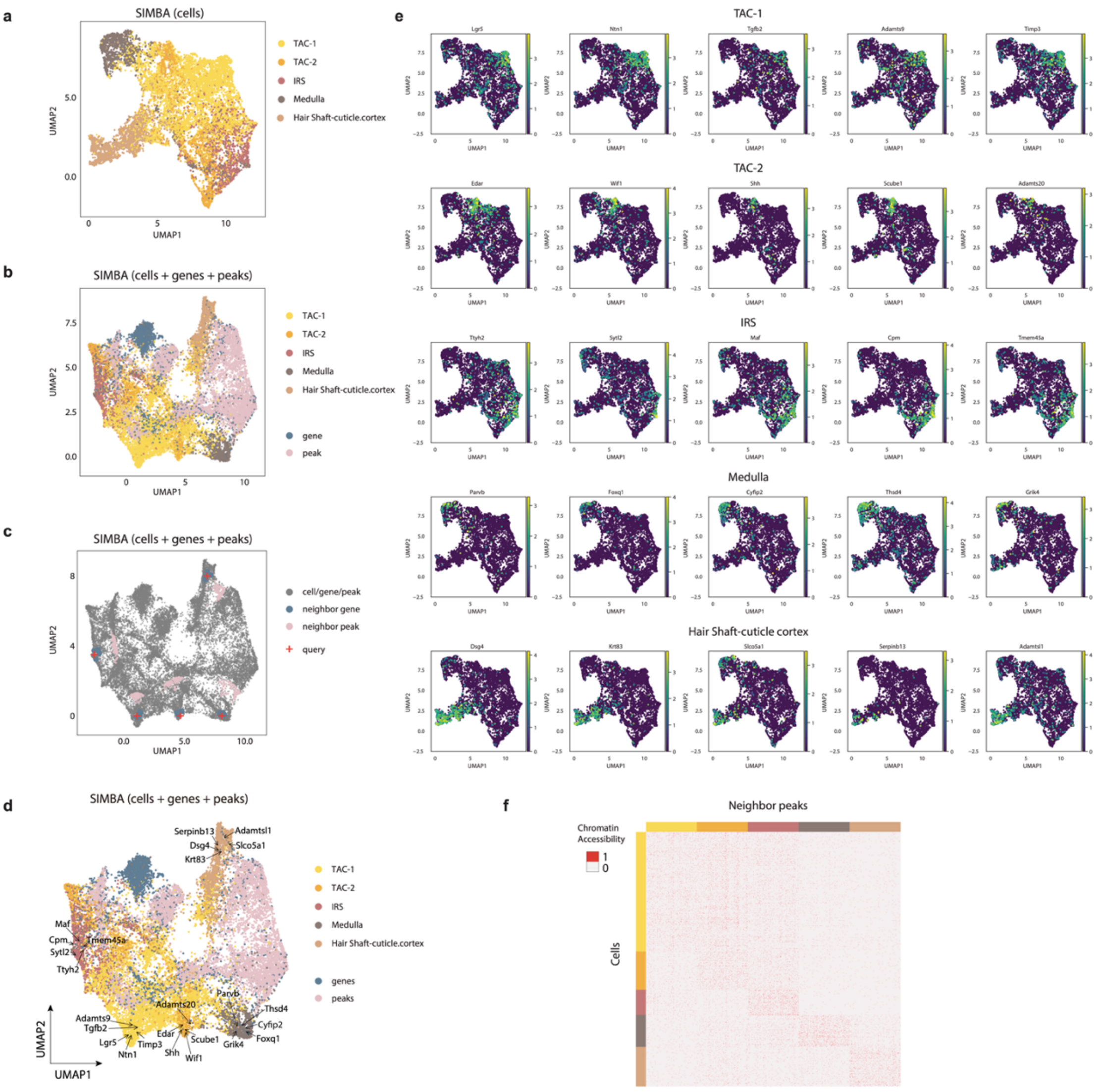

Supplementary Figure 21. SIMBA-inferred marker features for the SHARE-seq mouse skin dataset in multi-omics integration analysis.

- **a.** UMAP visualization of SIMBA embeddings of cells with two cellular modalities integrated.
- **b.** UMAP visualization of SIMBA embeddings of cells, genes, and peaks with two cellular modalities integrated.
- **c.** UMAP visualization of SIMBA embeddings of cells, genes, and peaks with two cellular modalities integrated. Biological “query” points are highlighted with a red “+”. Nearby informative genes and peaks are colored accordingly.
- **d.** UMAP visualization of SIMBA embeddings of cells, genes, and peaks with two cell modalities integrated and known marker genes highlighted.
- **e.** UMAP visualization of SIMBA embeddings of cells colored by indicated gene expression intensity, separated by cell type.
- **f.** Heatmap of cells against neighboring peaks of each cell type that are selected in the SIMBA co-embedding space. Chromatin accessibility is binary and colored accordingly.

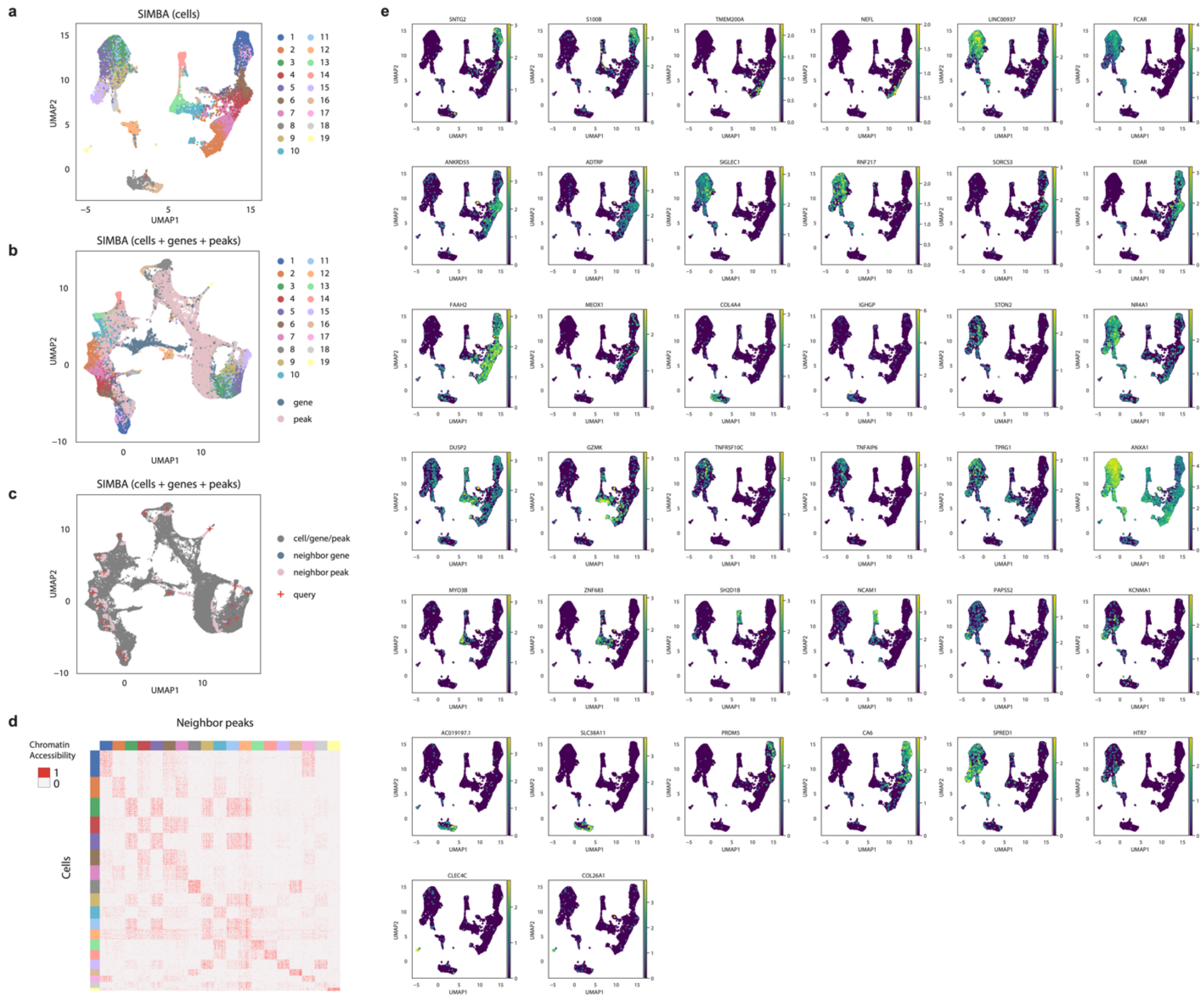

Supplementary Figure 22. SIMBA-inferred marker features for the 10x human PBMCs dataset in multi-omics integration analysis.

- **a.** UMAP visualization of SIMBA embeddings of cells with two cellular modalities integrated.
- **b.** UMAP visualization of SIMBA embeddings of cells, genes, and peaks with two cellular modalities integrated.
- **c.** UMAP visualization of SIMBA embeddings of cells, genes, and peaks with two cellular modalities integrated. Biological "query" points are highlighted with a red "+". Nearby informative genes and peaks are colored accordingly.
- **d.** Heatmap of cells against neighboring peaks of each cluster that are selected in the SIMBA co-embedding space. Chromatin accessibility is binary and colored accordingly.
- **e.** UMAP visualization of SIMBA embeddings of cells colored by indicated gene expression intensity, separated by cell type.

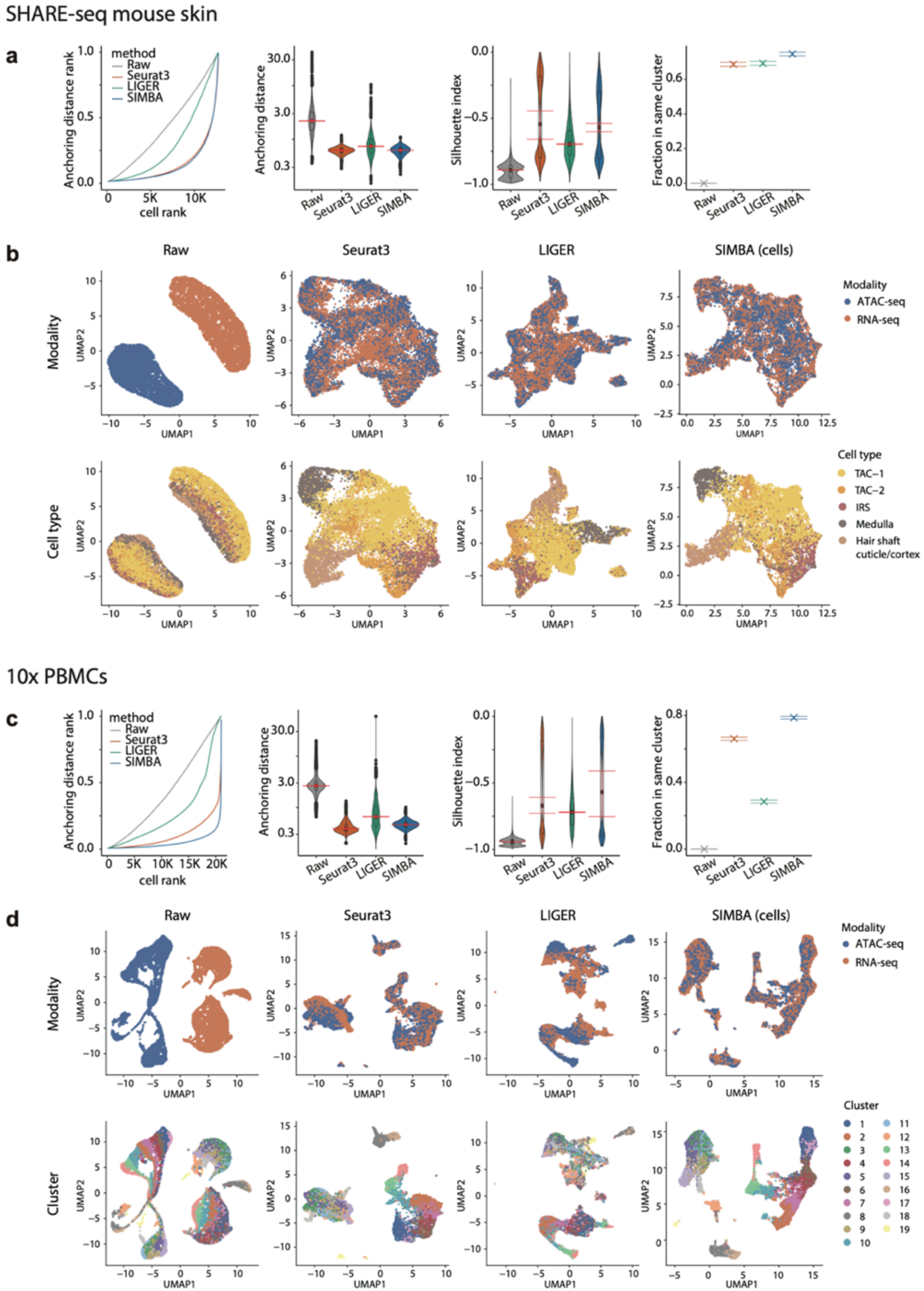
SHARE-seq mouse skin
a
method
Raw
Seurat3
LIGER
SIMBA
Anchoring distance rank
cell rank
Anchoring distance
Silhouette index
Fraction in same cluster
b
Raw
Seurat3
LIGER
SIMBA (cells)
Modality
ATAC-seq
RNA-seq
Cell type
TAC−1
TAC−2
IRS
Medulla
Hair shaft cuticle/cortex
UMAP1
UMAP2
10x PBMCs
c
method
Raw
Seurat3
LIGER
SIMBA
Anchoring distance rank
cell rank
Anchoring distance
Silhouette index
Fraction in same cluster
d
Raw
Seurat3
LIGER
SIMBA (cells)
Modality
ATAC-seq
RNA-seq
Cluster
UMAP1
UMAP2

Supplementary Figure 23. Comparison of SIMBA to other methods for multi-omics integration of the SHARE-seq mouse skin (a-b) and 10x multiome human PBMCs (c-d) datasets.

a, c. Quantitative comparison of SIMBA with two other methods including Seurat3, LIGER for multi-omics integration (n = 12,872 cells in **a**, n = 21,052 cells in **c**), using, left-to-right: anchoring distance rank, anchoring distance, silhouette index, and fraction in the same cluster. Boxes indicate first quarter, median, and third quarter of the data. The whiskers extend to $\pm$1.5 x the inter-quartile range (IQR). The red notches show a 95% confidence interval of the medians, which are calculated using a bootstrapping method with 3,000 iterations.

b, d. UMAP visualization of the raw scRNA-seq and scATAC-seq data from the 10x multiome human PBMCs dataset alongside the integrated results produced by Seurat3, LIGER, and SIMBA. Colored by data modality (top) and cluster assignment (bottom).

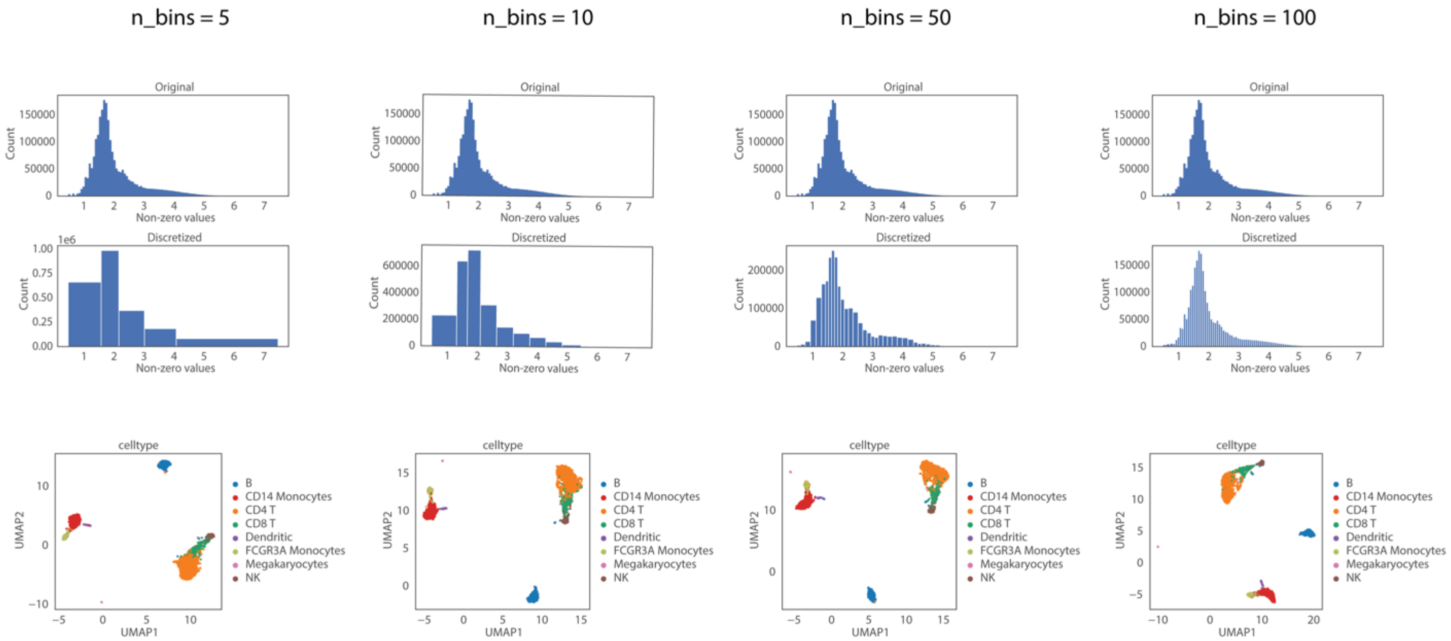

Supplementary Figure 24. Evaluation of the number of bins for SIMBA embeddings.
Top: The original distribution of non-zero gene expression values and the discretized distributions of non-zero gene expression values using different numbers of bins.
Bottom: UMAP visualization of SIMBA embeddings of cells generated using different number of bins on the 10x PBMCs dataset. Cells are colored by cell type.

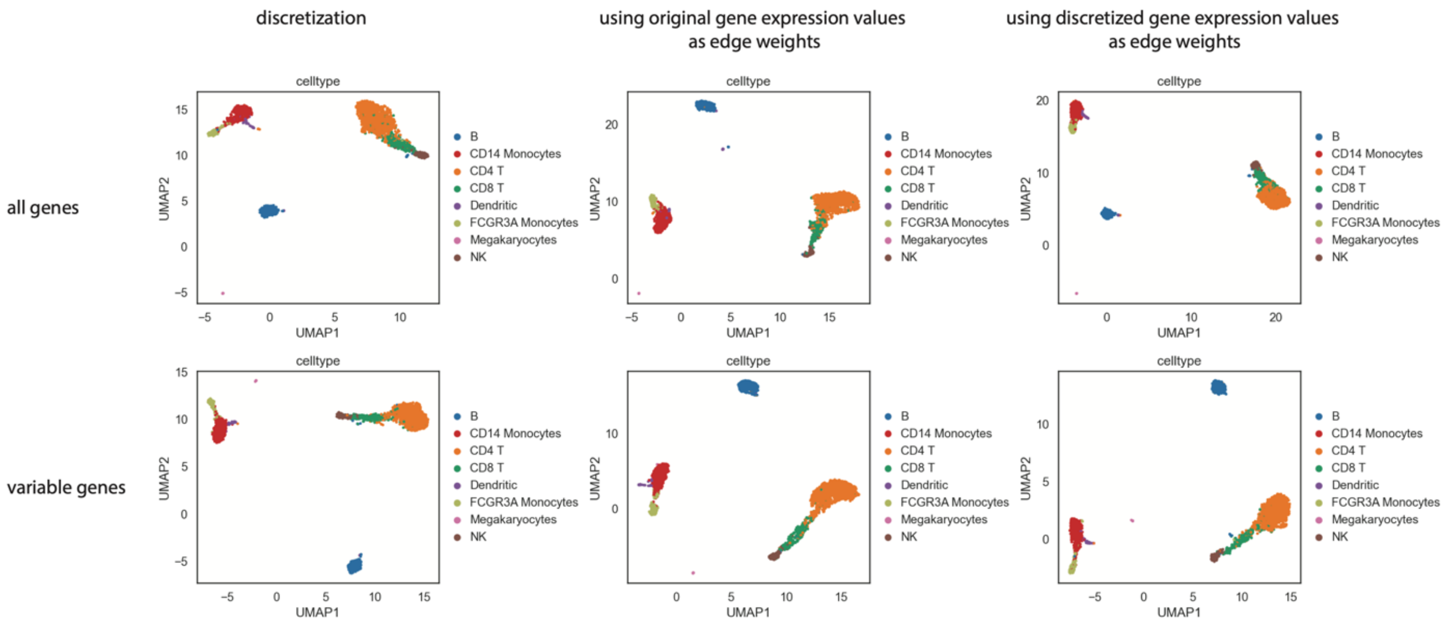

Supplementary Figure 25. Evaluation of edge weights for SIMBA embeddings.
UMAP visualization shows SIMBA embeddings of cells colored by cell type on the 10x PBMCs dataset. Each row of the panels represents the type of features used in SIMBA, including using all genes and using variable genes only. Each column of the panels represents the way the graph edges are weighted in SIMBA, including discretization, weighting edges using original gene expression, and weighting edges using discretized gene expression.

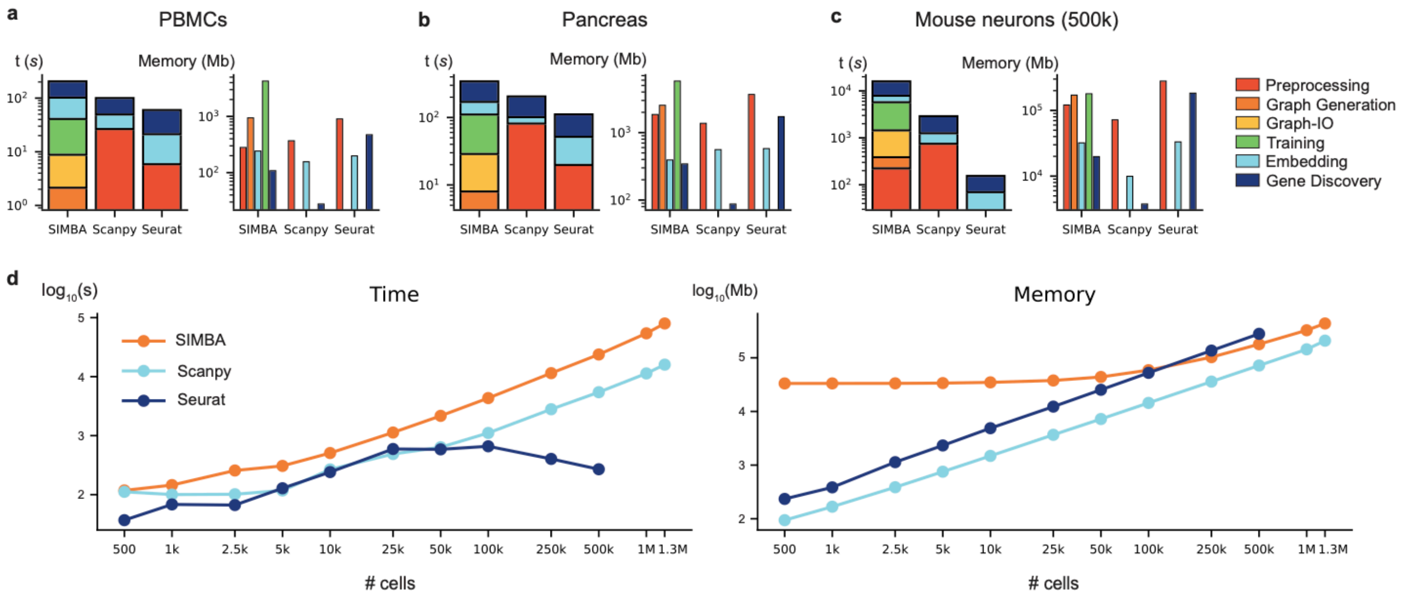

Supplementary Fig. 26. Benchmarking the computational complexity requirements of SIMBA against widely-used, state-of-the-art workflows for single-cell analysis in Python and R. Each plot shows the mean value of three iterations for SIMBA, Scanpy, and Seurat, categorized by functional contribution. a-c. Comparative run time and max memory consumption for each method as applied to (a) the 10x PBMCs (3,000 cell) dataset, (b) the Pancreas scRNA-seq (Baron et al., 2016) dataset, and (c) the 500,000 cell down-sampling of the 1.3M mouse neuron dataset (the largest down-sample that each method could run using default parameters). (d) Comparative run time and max memory consumption for each method applied to the 10x 1.3M cell mouse neuron dataset, progressively down-sampled from 500 cells to the full dataset.

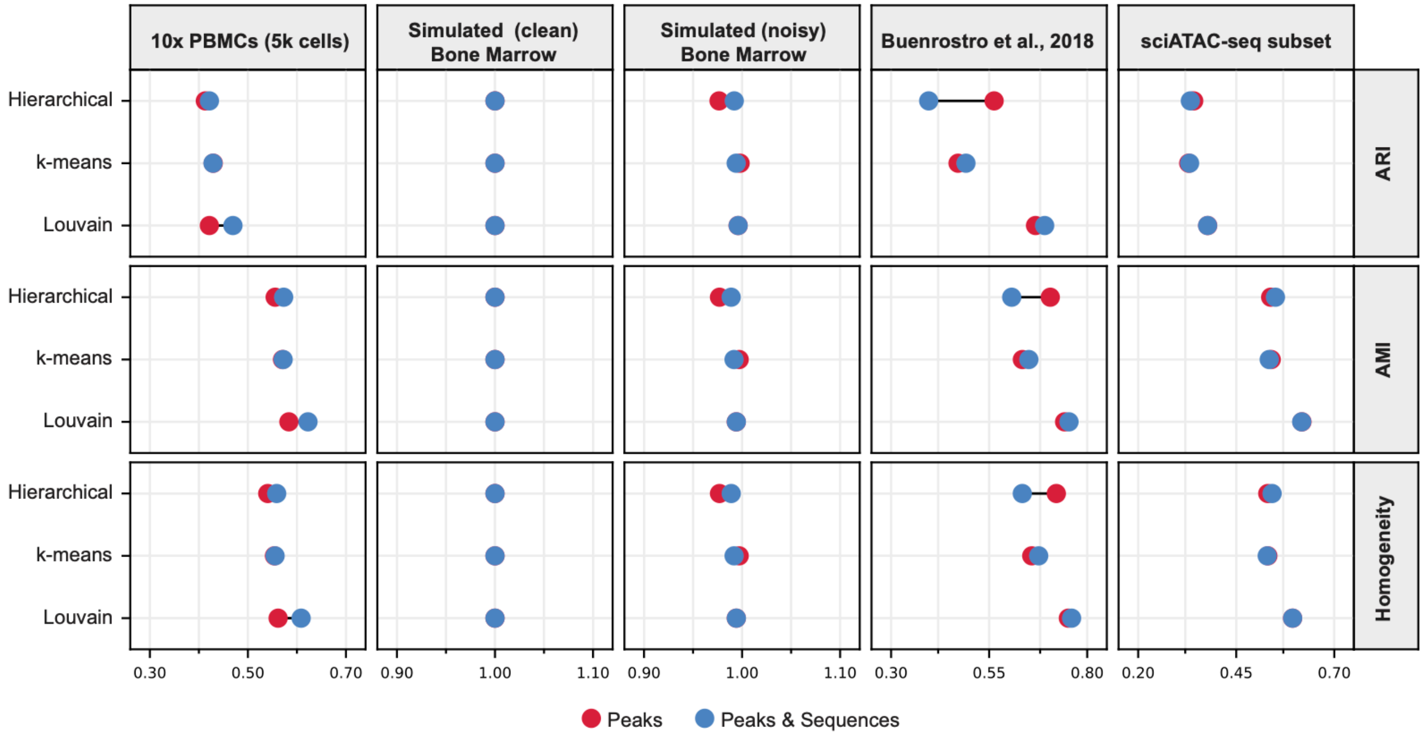

Supplementary Figure 27. Comparing the resulting SIMBA embedding via clustering solutions given additional genomic features. Each panel represents the combination of a dataset (columns) and a clustering evaluation metric (rows). Within each panel, the y-axis is organized according to the clustering algorithm applied and the x-axis describes the score achieved for the corresponding metric.

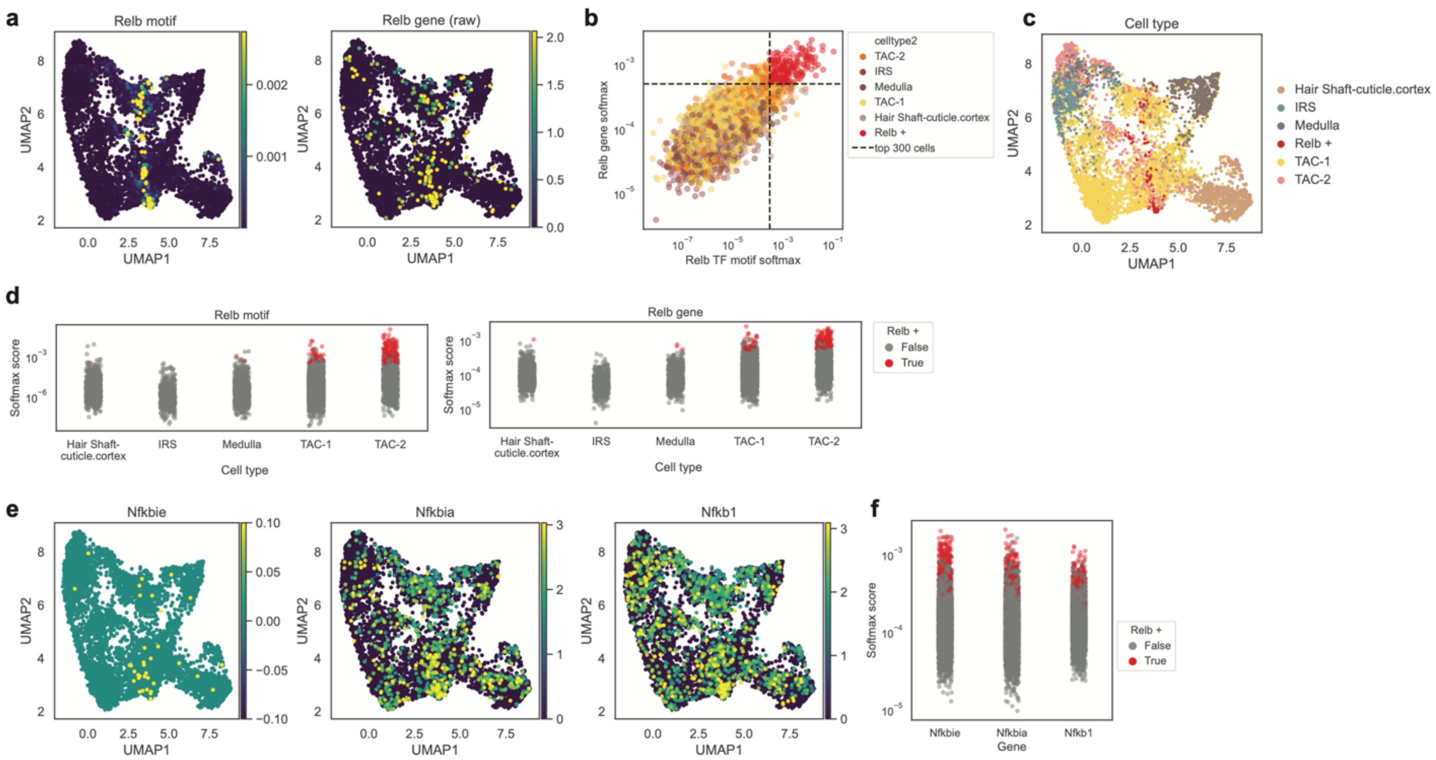

Supplementary Figure 28. Identification of the master regulator Relb and Relb+ cells on the mouse SHARE-seq dataset.

- **a.** UMAP visualization of SIMBA embeddings of cells colored by softmax probability scores of Relb motif and expression of Relb gene respectively.
- **b.** Scatterplot of Relb motif and Relb gene softmax probability scores of all cells. Top 300 cells are defined as Relb active ("Relb+") cells and are colored red.
- **c.** UMAP visualization of Relb+ cells along with other cell types defined in the original study.
- **d.** Stripplot of Relb motif and gene softmax probability scores for each cell type. Relb+ cells are colored red.
- **e.** UMAP visualization of SIMBA embeddings of cells colored by gene expression of the target genes identify by SIMBA, including *Nfkbie*, *Nfkbia*, and *Nfkb1*.
- **f.** Stripplot of softmax probability scores of *Nfkbie*, *Nfkbia*, and *Nkfb1* across all cells. Relb+ cells are colored red.

Supplementary Table 1: Comparison of SIMBA with existing methods

Method	Core algorithm	Graph nodes	Node featurization	Requires PCA or LSI as preprocessing steps	Output	scRNA-seq	scATAC-seq	Batch correction (single modality)	Multimodal (paired multi-omics)	Multi-omics integration (unpaired multi-omics)	Software Quality		
											Easy installation (software available in public packaging systems)	Dedicated Documentation website	Comprehensive and self-contained (no dependencies on heavy packages such as Scanpy or Seurat for preprocessing and plotting)
SIMBA	Multi-relation graph embeddings with type constraints	Cell and omics features (including genes, open regions, DNA sequences, etc.)	No	No	Co-embeddings of both cells and multiple features (comparable)	✓	✓	✓	✓	✓	✓	✓	✓
GLUE	Variational auto-encoders	Omics features (including genes, open regions, methylated sites)	No	Yes	Embeddings of cells and embeddings of multiple features (not comparable)	✗	✗	✗	✗	✓	✓	✓	✗
Metacell	k-nearest neighbors	Cells	Yes	Yes	Embeddings of cells	✓	✗	✗	✗	✗	✗	✗	✓
scETM * *not a graph-based method	Variational auto-encoders	NA	NA	No	Embeddings of cells and embeddings of single feature (genes) (not comparable)	✓	✗	✓	✗	✗	✓	✗	✗
CellSpace *not a graph-based method	TagSpace model (non-graphical embeddings) from Starspace	NA	NA	No	Co-embeddings of both cells and single feature (DNA sequences) (comparable)	✗	✓	✓	✗	✗	✗	✗	✗
MIRA	Variational autoencoder	cells	Yes	No	Embeddings of cells	✗	✗	✗	✓	✗	✓	✓	✗
