# Supplementary material for: SIMBA: single-cell embedding along with features
Source: Nat Methods. 2023 May 29;21(6):1003–13. doi: 10.1038/s41592-023-01899-8 (PMC11166568; doi:10.1038/s41592-023-01899-8)
Supplement: Supplementary file 2 — Reporting Summary [file 41592_2023_1899_MOESM2_ESM.pdf]

nature portfolio

Corresponding author(s): Luca Pinello
Adam Lerer

Last updated by author(s): Mar 6, 2023

Reporting Summary

Nature Portfolio wishes to improve the reproducibility of the work that we publish. This form provides structure for consistency and transparency in reporting. For further information on Nature Portfolio policies, see our Editorial Policies and the Editorial Policy Checklist.

Statistics

For all statistical analyses, confirm that the following items are present in the figure legend, table legend, main text, or Methods section.

n/a	Confirmed	
☐	☒	The exact sample size (*n*) for each experimental group/condition, given as a discrete number and unit of measurement
☒	☐	A statement on whether measurements were taken from distinct samples or whether the same sample was measured repeatedly
☐	☒	The statistical test(s) used AND whether they are one- or two-sided<br>*Only common tests should be described solely by name; describe more complex techniques in the Methods section.*
☐	☒	A description of all covariates tested
☐	☒	A description of any assumptions or corrections, such as tests of normality and adjustment for multiple comparisons
☐	☒	A full description of the statistical parameters including central tendency (e.g. means) or other basic estimates (e.g. regression coefficient) AND variation (e.g. standard deviation) or associated estimates of uncertainty (e.g. confidence intervals)
☐	☒	For null hypothesis testing, the test statistic (e.g. F, t, r) with confidence intervals, effect sizes, degrees of freedom and P value noted<br>*Give P values as exact values whenever suitable.*
☒	☐	For Bayesian analysis, information on the choice of priors and Markov chain Monte Carlo settings
☒	☐	For hierarchical and complex designs, identification of the appropriate level for tests and full reporting of outcomes
☒	☐	Estimates of effect sizes (e.g. Cohen's *d*, Pearson's *r*), indicating how they were calculated

Our web collection on statistics for biologists contains articles on many of the points above.

Software and code

Policy information about availability of computer code

Data collection	No software was used for data collection.

Data analysis

We provide a comprehensive Python package 'simba' available at https://anaconda.org/bioconda/simba and https://github.com/pinellolab/simba. All the proposed procedures are implemented in the "simba" package. 'simba' can be easily installed with conda "conda install simba". We also built a website (https://simba-bio.readthedocs.io), providing a detailed introduction of the 'simba' software and several SIMBA tutorials for different types of single-cell analyses presented in this manuscript. Scripts used for performance comparison are available at https://github.com/pinellolab/simba_comparison. The version of 'simba' used for the analyses presented in this manuscript was deposited at https://doi.org/10.5281/zenodo.7697337.

Tools used in the data analysis in this manuscript:

python v3.7
simba v1.1
simba_pbg v1.1
Scanpy v1.7.1
singleCellHaystack v0.3.4
CellID v 1.2.1
Seurat v3.2.3
LIGER v 0.5.0
Harmony v0.1.0

For manuscripts utilizing custom algorithms or software that are central to the research but not yet described in published literature, software must be made available to editors and reviewers. We strongly encourage code deposition in a community repository (e.g. GitHub). See the Nature Portfolio guidelines for submitting code & software for further information.

Data

Policy information about availability of data

All manuscripts must include a data availability statement. This statement should provide the following information, where applicable:

- Accession codes, unique identifiers, or web links for publicly available datasets
- A description of any restrictions on data availability
- For clinical datasets or third party data, please ensure that the statement adheres to our policy

All the datasets used in this study, including eight scRNA-seq datasets, four scATAC-seq datasets, and three dual-omics datasets are summarized in Supplementary Table 2 and are curated in the SIMBA package(https://simba-bio.readthedocs.io/en/latest/API.html#datasets). They can be easily downloaded and imported directly to reproduce the analyses presented in this manuscript. We have also deposited all the datasets to Zenodo at https://doi.org/10.5281/zenodo.7697355.

In addition, we also provide the source of these published datasets. For scRNA-seq datasets, the 10x PBMCs dataset is available at https://support.10xgenomics.com/single-cell-gene-expression/datasets/1.1.0/pbmc3k; the two mouse atlas datasets are available from https://github.com/JinmiaoChenLab/Batch-effect-removal-benchmarking/tree/master/Data/dataset2; the five human pancreas datasets are available from https://github.com/JinmiaoChenLab/Batch-effect-removal-benchmarking/tree/master/Data/dataset4. The scATAC-seq datasets are available from https://github.com/pinellolab/scATAC-benchmarking. For dual-omics datasets, the SHARE-seq mouse skin dataset is available from GSE140203; the mouse cerebral cortex SNARE-seq dataset is available from GSE126074; the 10 PBMCs multiome dataset is available from https://support.10xgenomics.com/single-cell-multiome-atac-gex/datasets/1.0.0/pbmc_granulocyte_sorted_10k.

Human research participants

Policy information about studies involving human research participants and Sex and Gender in Research.

Reporting on sex and gender	N/A
Population characteristics	N/A
Recruitment	N/A
Ethics oversight	N/A

Note that full information on the approval of the study protocol must also be provided in the manuscript.

Field-specific reporting

Please select the one below that is the best fit for your research. If you are not sure, read the appropriate sections before making your selection.

☒ Life sciences ☐ Behavioural & social sciences ☐ Ecological, evolutionary & environmental sciences

For a reference copy of the document with all sections, see nature.com/documents/nr-reporting-summary-flat.pdf

Life sciences study design

All studies must disclose on these points even when the disclosure is negative.

Sample size	A sample-size calculation was not conducted. Instead, the original authors who made their datasets publicly available determined the sample size.
Data exclusions	There was no exclusion of data.
Replication	We didn't have control over the experimental design because we utilized pre-existing public datasets. Thus, this is not applicable.
Randomization	We didn't have control over the experimental design because we utilized pre-existing public datasets. Thus, this is not applicable.
Blinding	We didn't have control over the experimental design because we utilized pre-existing public datasets. Thus, this is not applicable.

Reporting for specific materials, systems and methods

We require information from authors about some types of materials, experimental systems and methods used in many studies. Here, indicate whether each material, system or method listed is relevant to your study. If you are not sure if a list item applies to your research, read the appropriate section before selecting a response.

Materials & experimental systems

n/a	Involved in the study	
☒	☐	Antibodies
☒	☐	Eukaryotic cell lines
☒	☐	Palaeontology and archaeology
☒	☐	Animals and other organisms
☒	☐	Clinical data
☒	☐	Dual use research of concern

Methods

n/a	Involved in the study	
☒	☐	ChIP-seq
☒	☐	Flow cytometry
☒	☐	MRI-based neuroimaging
